# Structural basis of DNA methylation-dependent site selectivity of the Epstein–Barr virus lytic switch protein ZEBRA/Zta/BZLF1

**DOI:** 10.1093/nar/gkab1183

**Published:** 2021-12-10

**Authors:** Florent Bernaudat, Montse Gustems, Johannes Günther, Mizar F Oliva, Alexander Buschle, Christine Göbel, Priscilla Pagniez, Julien Lupo, Luca Signor, Christoph W Müller, Patrice Morand, Michael Sattler, Wolfgang Hammerschmidt, Carlo Petosa

**Affiliations:** Univ. Grenoble Alpes, CEA, CNRS, Institut de Biologie Structurale (IBS), 38000 Grenoble, France; European Synchrotron Radiation Facility, 71 avenue des Martyrs, 38043 Grenoble, France; Research Unit Gene Vectors, Helmholtz Zentrum München, German Research Center for Environmental Health, Munich, Germany and German Centre for Infection Research (DZIF), Partner site Munich, D-81377 Germany; Institute of Structural Biology, Helmholtz Center Munich, 85764 Neuherberg, Germany; Bavarian NMR Center and Department of Chemistry, Technical University of Munich, 85748 Gaching, Germany; Univ. Grenoble Alpes, CEA, CNRS, Institut de Biologie Structurale (IBS), 38000 Grenoble, France; Institut Laue-Langevin, 71 avenue des Martyrs, 38042 Cedex 9 Grenoble, France; Research Unit Gene Vectors, Helmholtz Zentrum München, German Research Center for Environmental Health, Munich, Germany and German Centre for Infection Research (DZIF), Partner site Munich, D-81377 Germany; Research Unit Gene Vectors, Helmholtz Zentrum München, German Research Center for Environmental Health, Munich, Germany and German Centre for Infection Research (DZIF), Partner site Munich, D-81377 Germany; Univ. Grenoble Alpes, CEA, CNRS, Institut de Biologie Structurale (IBS), 38000 Grenoble, France; Univ. Grenoble Alpes, CEA, CNRS, Institut de Biologie Structurale (IBS), 38000 Grenoble, France; Laboratoire de Virologie, Centre Hospitalier Universitaire Grenoble Alpes, 38000 Grenoble, France; Univ. Grenoble Alpes, CEA, CNRS, Institut de Biologie Structurale (IBS), 38000 Grenoble, France; Structural and Computational Biology Unit, European Molecular Biology Laboratory (EMBL), D-69117 Heidelberg, Germany; Univ. Grenoble Alpes, CEA, CNRS, Institut de Biologie Structurale (IBS), 38000 Grenoble, France; Laboratoire de Virologie, Centre Hospitalier Universitaire Grenoble Alpes, 38000 Grenoble, France; Institute of Structural Biology, Helmholtz Center Munich, 85764 Neuherberg, Germany; Bavarian NMR Center and Department of Chemistry, Technical University of Munich, 85748 Gaching, Germany; Research Unit Gene Vectors, Helmholtz Zentrum München, German Research Center for Environmental Health, Munich, Germany and German Centre for Infection Research (DZIF), Partner site Munich, D-81377 Germany; Univ. Grenoble Alpes, CEA, CNRS, Institut de Biologie Structurale (IBS), 38000 Grenoble, France

## Abstract

In infected cells, Epstein–Barr virus (EBV) alternates between latency and lytic replication. The viral bZIP transcription factor ZEBRA (Zta, BZLF1) regulates this cycle by binding to two classes of ZEBRA response elements (ZREs): CpG-free motifs resembling the consensus AP-1 site recognized by cellular bZIP proteins and CpG-containing motifs that are selectively bound by ZEBRA upon cytosine methylation. We report structural and mutational analysis of ZEBRA bound to a CpG-methylated ZRE (meZRE) from a viral lytic promoter. ZEBRA recognizes the CpG methylation marks through a ZEBRA-specific serine and a methylcytosine-arginine-guanine triad resembling that found in canonical methyl-CpG binding proteins. ZEBRA preferentially binds the meZRE over the AP-1 site but mutating the ZEBRA-specific serine to alanine inverts this selectivity and abrogates viral replication. Our findings elucidate a DNA methylation-dependent switch in ZEBRA’s transactivation function that enables ZEBRA to bind AP-1 sites and promote viral latency early during infection and subsequently, under appropriate conditions, to trigger EBV lytic replication by binding meZREs.

## INTRODUCTION

DNA methylation in mammals is a major epigenetic modification that primarily occurs at the cytosine C5 position within CpG motifs (1). DNA methylation is commonly perceived as a repressive epigenetic mark that induces transcriptional silencing. Silencing is mediated by methyl-CpG binding proteins (MBPs) that inhibit the action of RNA polymerase II or lead to a restrictive chromatin state (2,3). In addition, CpG methylation can directly inhibit the binding of transcription factors to their DNA target sites (4,5). On the contrary, the expression of certain genes may be enhanced by DNA methylation, and a growing number of transcription factors are known to display a preference for methylated target sequences (6–13). The first of these to be identified was the EBV protein ZEBRA (also called BZLF1, Zta, Z or EB1) (6).

DNA methylation plays a pivotal role in the EBV infection cycle (14). EBV is a gamma herpesvirus that infects >90% of the world population, can cause Infectious Mononucleosis in adolescents and young adults and is associated with several epithelial and B-cell malignancies (15). EBV primarily infects B lymphocytes and has a biphasic infection cycle that alternates between latency and lytic replication (16). Upon infection, during a stage termed prelatency (17), EBV delivers its linear genomic DNA to the host cell nucleus, where multiple copies of the viral genome are maintained as plasmids (also termed episomes) that are initially unmethylated. During prelatency the viral genome becomes chromatinized, histones acquire post-translational modifications, and the viral DNA becomes progressively methylated at CpG motifs (18). These changes allow EBV to repress the expression of immunodominant viral antigens and establish a strictly latent infection in memory B lymphocytes, thereby evading host immune surveillance (19). Antigen-mediated stimulation of the B-cell receptor signaling pathway can reactivate the virus in plasma cells *in vivo* (20) and induce a cascade of immediate-early, early and late lytic gene expression, leading to viral *de novo* synthesis and release of progeny. Evidence suggests that EBV lytic replication contributes to lymphomagenesis (21–23).

**Figure 1.**
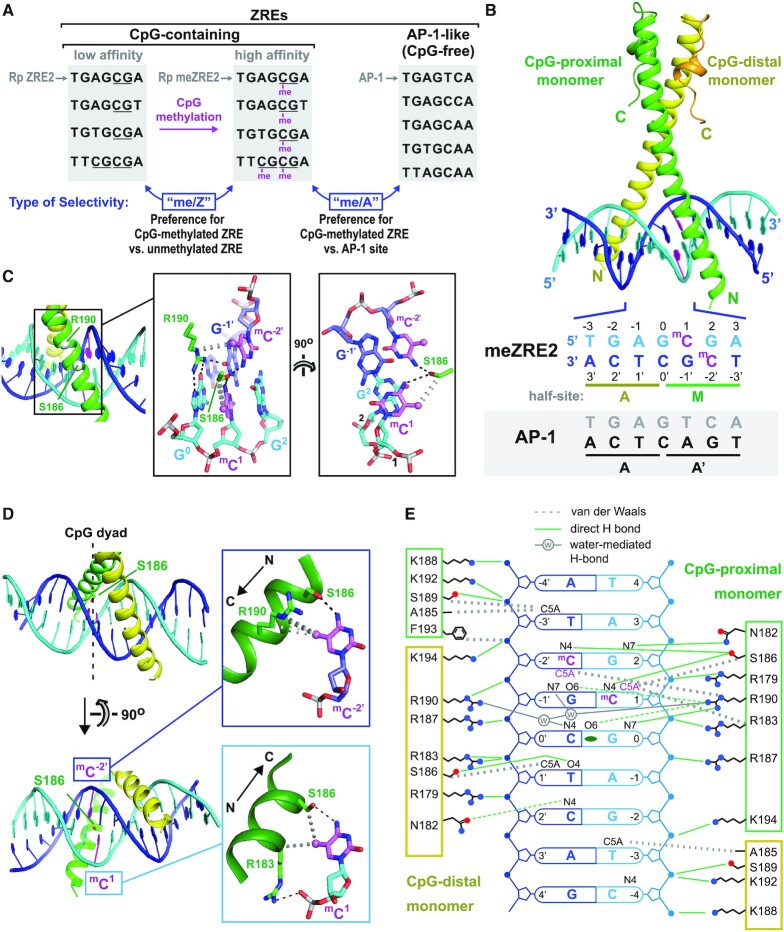
Structure of ZEBRA bound to meZRE2. (**A**) Representative ZRE sites. CpG-containing sites are (from top to bottom): Rp ZRE2, Nap ZRE2, BMRF1(−248) and Rp ZRE3 (6,28,35). AP-1-like sites are AP-1, Zp ZREIIIA, DSL ZRE7, DSL ZRE5 and Zp ZREIIIB (24,30-32). (**B**) Structure of ZEBRA’s DNA-binding domain in complex with meZRE2. Methylcytosine bases are in magenta. The AP-1 sequence is included for comparison. The A half-site, which includes G^0^, is common to both the AP-1 and meZRE2 sites. Note that the structure closely resembles that of the ZEBRA/meZRE2 complex previously reported by Hong *et al.* (42). (**C**) Details of the ^m^CpG site showing interactions involving Ser^186^ and Arg^190^. Black and gray dashed lines indicate H-bonds and van der Waals contacts, respectively. (**D**) The Ser^186^ side chain is positioned directly on the local dyad relating the two antisense ^m^CpG dinucleotides. Top inset: ZEBRA recognizes the ^m^C^–2′^ methyl group through van der Waals contacts with the Arg^190^ side chain. Bottom inset: ZEBRA senses the methyl group on ^m^C^1^ through van der Waals contacts with the Arg^183^ backbone Cα atom and the Ser^186^ side chain methylene group. (**E**) Schematic summary of protein–DNA interactions. Residues in the CpG-distal and -proximal monomers are enclosed in yellow and green boxes, respectively. Green and gray lines represent direct and water-mediated H-bonds, respectively. A weak H-bond formed by CpG-distal Asn^182^ and bifurcated H-bonds formed by CpG-proximal Arg^190^ are shown as broken green lines. Dotted gray lines indicate van der Waals contacts. Protein side chains are illustrated with oxygen and nitrogen atoms shown as red and blue dots, respectively.

ZEBRA is a homodimeric protein related to the activating protein 1 (AP-1) family of bZIP transcription factors (24). ZEBRA regulates the EBV infection cycle by playing key roles both in establishing viral latency and triggering lytic replication. The transient expression of ZEBRA during prelatency when the EBV genome is unmethylated is critical for promoting the proliferation of quiescent naive and memory B cells that favors the transition to strict latency (18). During latency, when the EBV genome is methylated, the expression of ZEBRA activates a second viral transcription factor, Rta, which acts together with ZEBRA to trigger lytic replication (25,26). Underpinning ZEBRA’s dual role in prelatency and lytic activation is its ability to recognize two distinct classes of DNA target sites, collectively termed ZEBRA responsive elements (ZREs) (27,28) (Figure 1A). One class comprises viral and cellular sites resembling the AP-1 consensus sequence TGAGTCA [also called TPA responsive element (TRE) (29)] recognized by cellular AP-1 proteins (24,30–32). The binding of ZEBRA to cellular AP-1 sites during prelatency is critical for promoting the proliferation of EBV-infected resting B cells (18,33). The second class comprises CpG-containing sites with the consensus TGAGCGA, which ZEBRA selectively binds when methylated. Many lytic EBV promoters have CpG-containing ZREs whose binding by ZEBRA is methylation dependent, including the Rta promoter (Rp) (6,18,27–28,28,34–36). Moreover, ZEBRA behaves like a pioneer transcription factor (PTF) that can directly bind nucleosomal DNA, recruit chromatin remodelers and enhance the local accessibility of chromatin (37). Thus, whereas host-driven methylation of the EBV genome ordinarily represses viral gene expression, ZEBRA’s PTF-like behavior and ability to activate CpG-methylated viral lytic promoters allow it to overturn host-mediated epigenetic silencing.

ZEBRA’s ability to preferentially bind and activate methylated viral promoters is attributed to a serine residue in its bZIP domain, Ser^186^, that differs conspicuously from the corresponding alanine conserved in cellular bZIP proteins (34). Alanine substitution of Ser^186^ compromises ZEBRA’s ability to bind methylated ZREs and activate viral early lytic genes (34,38–42). Conversely, Ala→Ser mutations of the corresponding residues enabled a heterodimer of the cellular AP-1 proteins Fos and Jun to acquire these activities (41). The crystal structure of ZEBRA’s DNA-binding domain in complex with the consensus AP-1 site revealed the unusual bZIP fold of this domain and the details of AP-1 site recognition (43). A subsequent structural study revealed how ZEBRA achieves methylated ZRE (meZRE) recognition, including a direct contact between Ser^186^ and the ^m^CpG motif, and related these findings to how a Jun homodimer recognizes a methylated AP-1 site (42). Recently, an additional basic motif upstream of the bZIP domain was shown to enhance the affinity of ZEBRA for a meZRE and to be required for late viral lytic gene expression (44).

Despite important advances in our understanding of ZEBRA structure and function, the molecular basis and functional implications of ZEBRA’s dual specificity for AP-1 and CpG-methylated sites remain poorly understood. Here, we analyze the structure of ZEBRA bound to a CpG-methylated ZRE in a detailed comparison with the AP-1-bound structure. We investigate how the integrity of interactions that mediate ^m^CpG recognition correlate with ZEBRA’s functions to transactivate a methylated promoter and to activate viral lytic replication. We found that several distinct DNA-contacting residues are required for both these activities. Surprisingly, most of these residues, in particular Ser^186^, contribute only to a limited degree to ZEBRA’s ability to discriminate between the methylated and unmethylated forms of a CpG-containing ZRE. In striking contrast, Ser^186^ is the critical determinant for ZEBRA’s preference to bind a CpG-methylated ZRE versus an AP-1 sequence motif. Our data document that ZEBRA’s DNA-binding interface is fine-tuned to discriminate between its two classes of ZRE target sites rather than simply to differentiate methylated from unmethylated DNA. More generally, our findings reveal how ZEBRA is capable of switching between AP-1 and CpG-methylated sites, enabling EBV’s biphasic life style to establish latency upon infection and to escape from it, eventually.

## MATERIALS AND METHODS

### Protein expression and purification

For structure determination we used a ZEBRA construct spanning residues 175–236, which lacks the 9 C-terminal residues (res. 237–245) previously shown to reduce solubility (43) and replaced residue Cys^189^ by a serine. The C189S mutation does not alter ZEBRA’s binding affinity toward the meZRE2 site (Figure 5B and Table 1) but was critical for obtaining well-diffracting crystals. A pET28a vector expressing this ZEBRA construct was used to transform *Escherichia coli* strain BL21 (DE3). Cells were grown in LB medium containing kanamycin (60 μg/ml) until an OD_600_ of 0.8 was reached. Expression was induced with 0.5 mM isopropyl β-D-thiogalactopyranoside (IPTG) and cells further incubated at 37°C for 5 h. Harvested cells were lysed by sonication in lysis buffer [10 mM NaCl, 20 mM TRIS/HCl pH 6.8, 5 mM β-mercaptoethanol, 1 mM PMSF, cOmplete EDTA-free (Boehringer, 1 tablet/50 ml)]. Nucleic acids were removed from the cleared lysate by polyethyleneimine (PEI) precipitation (0.3% v/v). The protein was purified by SP Sepharose chromatography (GE Healthcare) in lysis buffer using a 0.01–1 M NaCl gradient, followed by ammonium sulfate precipitation (30% w/v) and Superdex 75 chromatography (GE Healthcare) in 20 mM TRIS/HCl (pH 7.5), 150 mM NaCl, 150 mM ammonium acetate, 5 mM DTT and 0.2 mM PMSF.

**Table 1.** Summary of binding data

**Methylated versus unmethylated sites**							
**Protein**		**DNA**	***K*_d, app_ (nM)**	**Selectivity relative** **to ZRE2^1,2^**	**Δ*G*_app_ (kcal/mol)^1^**	**ΔΔ*G*_app_ versus ZRE2** **(ΔΔ*G*_me/Z_ values in bold)** **(kcal/mol)^1,3^**	**ΔΔ*G*_me/A_ (kcal/mol)^1,4^**
ZEBRA	WT	ZRE2	1710 ± 285		−7.86 ± 0.10		
		hemi(C^-2′^)	667 ± 147	2.6 ± 1.0	−8.42 ± 0.14	−0.56 ± 0.24	
		hemi(C^1^)	167 ± 34	10.2 ± 3.8	−9.24 ± 0.13	−1.38 ± 0.23	
		meZRE2	75.1 ± 19.8	22.8 ± 9.8	−9.71 ± 0.17	−**1.85 ± 0.27**	−0.76 ± 0.23
		AP-1	271 ± 25	6.3 ± 1.6	−8.95 ± 0.06	−1.09 ± 0.16	
	S186T	ZRE2	2990 ± 208		−7.53 ± 0.04		
		meZRE2	121 ± 44	24.7 ± 10.7	−9.43 ± 0.26	−**1.90 ± 0.30**	
	S186A	ZRE2	5210 ± 1010		−7.20 ± 0.12		
		meZRE2	434 ± 28	12.0 ± 3.1	−8.68 ± 0.04	−**1.48 ± 0.16**	0.69 ± 0.17
		AP-1	136 ± 29	38.3 ± 15.6	−9.36 ± 0.13	−2.16 ± 0.25	
	S186C	ZRE2	7730 ± 1650		−6.97 ± 0.14		
		meZRE2	1250 ± 264	6.2 ± 2.6	−8.05 ± 0.13	−**1.08 ± 0.27**	
	C189S	ZRE2	1330 ± 190		−8.01 ± 0.09		
		meZRE2	87.3 ± 17.5	15.2 ± 5.2	−9.62 ± 0.13	−**1.61 ± 0.22**	
	C189A	ZRE2	2360 ± 453		−7.67 ± 0.12		
		meZRE2	118 ± 19	20.0 ± 7.1	−9.45 ± 0.10	−**1.78 ± 0.22**	
	C189T	ZRE2	2470 ± 258		−7.65 ± 0.06		
		meZRE2	171 ± 22	14.4 ± 3.4	−9.23 ± 0.08	−**1.58 ± 0.14**	
	N182A	ZRE2	1850 ± 313		−7.82 ± 0.10		
		meZRE2	85.3 ± 14.5	21.7 ± 7.4	−9.64 ± 0.11	−**1.82 ± 0.21**	
	R183A	ZRE2	2390 ± 434		−7.67 ± 0.11		
		meZRE2	162 ± 34	14.8 ± 5.8	−9.26 ± 0.13	−**1.59 ± 0.24**	
	R190A	ZRE2	5350 ± 841		−7.19 ± 0.10		
		meZRE2	1310 ± 159	4.1 ± 1.1	−8.02 ± 0.07	−**0.83 ± 0.17**	
GCN4	WT	ZRE2	3940 ± 1080		−7.37 ± 0.18		
		meZRE2	479 ± 41	8.2 ± 3.0	−8.62 ± 0.05	−**1.25 ± 0.23**	1.08 ± 0.12
		AP-1	77.9 ± 8.8	50.6 ± 19.5	−9.69 ± 0.07	−2.32 ± 0.25	
	A239S	ZRE2	1600 ± 460		−7.90 ± 0.19		
		meZRE2	98.0 ± 15.6	16.3 ± 7.3	−9.56 ± 0.10	−**1.66 ± 0.29**	−0.32 ± 0.18
		AP-1	169 ± 22	9.5 ± 4.0	−9.23 ± 0.08	−1.33 ± 0.27	
**Hydroxymethylated versus methylated ZRE2**							
**Protein**		**DNA**	***K*_d, app_ (nM)**	**Selectivity of meZRE2 relative** **to site** ^5^	**Δ*G*_app_ (kcal/mol)**	**ΔΔ*G*_app_** **versus meZRE2** **(kcal/mol)^6^**	
ZEBRA		meZRE2	69 ± 13		−9.76 ± 0.12		
		hemi(^hm^C^1^)	113 ± 14	1.6 ± 0.5	−9.47 ± 0.08	0.29 ± 0.19	
		hemi(^hm^C^–2′^)	114 ± 18	1.6 ± 0.6	−9.47 ± 0.10	0.30 ± 0.21	
		hmZRE2	216 ± 23	3.1 ± 0.9	−9.09 ± 0.06	0.68 ± 0.18	

^1^Data shown represent the mean values ± SD from three independent experiments.

^2^Calculated as *K*_1_/*K*_2_, where *K*_1_ is the *K*_d, app_ for ZRE2 and *K*_2_ is that for the compared site. The error was determined as ϵ = (ϵ_1_/*K*_1_ + ϵ_2_/*K*_2_)*(*K*_1_/*K*_2_), where ϵ_1_ and ϵ_2_ are the errors associated with *K*_1_ and *K*_2_.

^3^Calculated as Δ*G*_2_ – Δ*G*_1_, where Δ*G*_1_ is the Δ*G*_app_ value for ZRE2 and Δ*G*_2_ is that of the compared site. The error was determined as ϵ = ϵ_1_ + ϵ_2_, where ϵ_1_ and ϵ_2_ are the errors associated with Δ*G*_1_ and Δ*G*_2_, respectively. ΔΔ*G*_app_ is identical to ΔΔ*G*_me/Z_ when the compared site is meZRE2.

^4^Calculated as Δ*G*_2_ – Δ*G*_1_, where Δ*G*_1_ is the Δ*G*_app_ value for AP-1 and Δ*G*_2_ is that for meZRE2. The error was determined as ϵ = ϵ_1_ + ϵ_2_, where ϵ_1_ and ϵ_2_ are the errors associated with Δ*G*_1_ and Δ*G*_2_, respectively.

^5^Calculated as *K*_1_/*K*_2_, where *K*_2_ is the *K*_d, app_ for meZRE2 and *K*_1_ is that for the compared site. The error was determined as ϵ = (ϵ_1_/*K*_1_ + ϵ_2_/*K*_2_)*(*K*_1_/*K*_2_), where ϵ_1_ and ϵ_2_ are the errors associated with *K*_1_ and *K*_2_.

^6^Calculated as Δ*G*_2_ – Δ*G*_1_, where Δ*G*_2_ is the Δ*G*_app_ value for meZRE2 and Δ*G*_1_ is that of the compared site.

MBP-tagged constructs of the DNA-binding domains of ZEBRA (res. 175–236) and *Saccharomyces cerevisiae* GCN4 (res. 228–281) used for FP assays were expressed from a pET-M40 vector in *E. coli* strain BL21-CodonPlus (DE3)-RIL. Cells were sonicated in lysis buffer (100 mM NaCl, 20 mM TRIS/HCl pH 7.2, 5 mM β-mercaptoethanol, 1 mM PMSF, EDTA-free cOmplete inhibitor [Boehringer] 1 tablet/50 ml). The cleared lysate was incubated with amylose resin (New England Biolabs) pre-equilibrated in lysis buffer and extensively washed with high-salt buffer (1.5 M NaCl, 20 mM TRIS/HCl pH 7.2, 5 mM β-mercaptoethanol). MBP-tagged proteins were eluted with high-salt buffer containing 10 mM maltose and further purified by Superdex 200 10/300 chromatography (GE Healthcare) in phosphate-buffered saline (137 mM NaCl, 2.7 mM KCl, 10 mM Na_2_HPO_4_, 1.8 mM KH_2_PO_4_, pH 7.4).

### Crystallization and structure determination

DNA oligonucleotides used for crystallization (5′-AAGCACTGAG(^m^C)GATGAAGT-3′ and 5′-TACTTCAT(^m^C)GCTCAGTGCT-3′) were chemically synthesized (Eurofins MWG) and subsequently purified by anion-exchange chromatography using a monoQ HR10/10 (GE Healthcare) column pre-equilibrated in 10 mM NaOH. Oligonucleotides were eluted using a linear NaCl gradient (0–1 M), dialysed against deionized water, lyophilized and subsequently dissolved in deionized water. Equimolar amounts of complementary oligonucleotides were mixed at a concentration of 10 mg/ml in 0.1 M NaCl, 10 mM HEPES pH 7.5, 10 mM MgCl_2_, 1 mM DTT and annealed in a water bath by cooling from 90 to 20°C over several hours. Hanging drop crystallization trials were carried out at 20°C by mixing equal volumes of reservoir solution and an equimolar protein:DNA mixture. Crystals grew from 22% polyethylene glycol (PEG) 4K, 18% PEG 400, 50 mM sodium acetate pH 4 and 20% isopropanol.

Diffraction data were collected from crystals flash cooled in liquid nitrogen at ESRF beamline ID23-2 (λ = 0.873 Å) on a MAR CCD 165 mm detector. Data were processed with XDS (45) and programs of the CCP4 suite (46). Molecular replacement was performed with Phaser (47) and the structure was refined with Phenix (48). Crystals contain two protein/DNA complexes in the asymmetric unit. The electron density is well defined for Complex 1 (chains A-D) but considerably poorer for Complex 2 (chains E-H), which exhibits high B factors and two-fold disorder around the DNA pseudodyad due to a lack of stabilizing crystallization contacts, explaining why *R*_cryst_ and *R*_free_ values are higher than those normally expected at this resolution. The accuracy of the structure is supported by stereochemical quality criteria (Supplementary Table S1), a high correlation coefficient (CC) with the local electron density for most residues (overall CC is 0.92 for complex 1 and 0.88 for complex 2) and low RMSD values with previously reported ZEBRA structures (Supplementary Figure S1). DNA geometry was analysed using the program 3DNA (49).

### Fluorescence polarization (FP) DNA-binding assay

The following pairs of oligonucleotides were chemically synthesized (Eurofins MWG) for FP assays involving (i) the AP-1 site: 5′-AATAAAATGACTCATAAGC-3′ and Rho-5′-AGCTTATGAGTCATTTTAT-3′ and (ii) the unmethylated, hemi-methylated and fully methylated ZRE2 sites: 5′-AATAAAATXGCTCATAAGC-3′ and Rho-5′-AGCTTATGAGXGATTTTAT-3′ where X represents either C or ^m^C and Rho represents the rhodamine label. Complementary oligonucleotides were dissolved in 0.15 M NaCl, 10 mM TRIS/HCl pH 7.5, 1 mM EDTA and annealed in a PCR machine. MBP-ZEBRA was serially diluted in phosphate-buffered saline (137 mM NaCl, 2.7 mM KCl, 10 mM Na_2_HPO_4_, 1.8 mM KH_2_PO_4_, pH 7.4) containing rhodamine-labeled duplex DNA (10 nM) and unlabeled herring testes Type XIV DNA (28 ng/μl) (SIGMA D6898) and incubated in a volume of 40 μl for 30 min in a 384-well plate. Fluorescence polarization was measured at 20°C using a SYNERGY 4 plate reader (BioTek). Excitation and emission wavelengths were 530 and 580 nm, respectively, and the slit width was 5 nm in both cases. Between two and four independent experiments (three technical replicates per experiment) were performed for each protein/DNA combination. Data were fitted as *FP* = *FP*_min_+(*FP*_max_ - *FP*_min_)*c^*n*^/(*c^n^* + *K*_d,app_^*n*^), where *FP*_min_ and *FP*_max_ are the lower baseline and upper plateau values of FP, and *c* is the total protein concentration. The Hill coefficient, *n*, was set at 2, consistent with empirical values of *n* derived from Hill plots that varied between 1.5 and 2.5 and in agreement with previous DNA-binding studies of bZIP proteins performed in the presence of non-specific competitor DNA (50,51). Binding curves for assays involving unmethylated ZRE2 where saturation was not fully attained could be reliably fitted because of highly reproducible values of *FP*_max_ across the ensemble of assays, and in several cases the results were confirmed by performing single-replicate experiments using higher protein concentrations.

### LC/ESI mass spectrometry

Liquid chromatography electrospray ionization mass spectrometry (LC/ESI-MS) was performed on a 6210 LC-TOF spectrometer coupled to a HPLC system (Agilent Technologies). All solvents used were HPLC grade (Chromasolv, Sigma-Aldrich), trifluoroacetic acid (TFA) was from Acros Organics (puriss., p.a.). Solvent A was 0.03% TFA in water; solvent B was 95% acetonitrile-5% water-0.03% TFA. Just before analysis, MBP-ZEBRA samples (10 μM in phosphate-buffered saline: 137 mM NaCl, 2.7 mM KCl, 10 mM Na_2_HPO_4_, 1.8 mM KH_2_PO_4_, pH 7.4) containing 0 or 20 mM DTT were diluted to a final concentration of 5 μM with water and 4 μl were injected for MS analysis. Protein samples were first desalted on a reverse phase-C8 cartridge (Zorbax 300SB-C8, 5 μm, 300 μm ID × 5 mm, Agilent Technologies) for 3 min at a flow rate of 50 μl/min with 100% solvent A and then eluted with 70% solvent B at flow rate of 50 μl/min for MS detection. MS acquisition was carried out in the positive ion mode in the 300–3200 *m/z* range. MS spectra were acquired and the data processed with MassHunter workstation software (v. B.02.00, Agilent Technologies) and GPMAW software (v. 7.00b2, Lighthouse Data, Denmark).

### NMR

DNA oligonucleotides were purchased (Eurofins MWG) and dissolved in water. DNA duplexes were prepared by mixing both strands in equimolar amounts. For this, DNA was heated to 95°C for 5 min and then slowly cooled at RT for at least 30 min. After lyophilization, the DNA was reconstituted in a buffer containing 50 mM sodium phosphate pH 6.5, 100 mM NaCl, and 10% D_2_O with a final concentration of 400 μM duplex DNA. Homonuclear ^1^H,^1^H NOESY experiments using water-flipback combined with WATERGATE for solvent suppression were carried out at 293K on a Bruker 950 MHz spectrometer equipped with z-gradient triple resonance cryoprobe. Spectra were processed using TopSpin (Bruker) and analyzed using the CCPN software suite (52).

### Cells

HEK293 and Raji cells were maintained in RPMI 1640 medium with 10% fetal calf serum (FCS), 1% penicillin–streptomycin and 1% sodium pyruvate at 37°C and 5% CO_2_. The ZEBRA knockout producer cell line 6169 contains an EBV genome based on the wt/B95.8 strain termed r_wt/B95.8 (6008) (53) with a stop codon after amino acid 56 of ZEBRA. The ZEBRA knockout EBV producer cells were maintained in RPMI 1640 medium with 10% FCS, 1% penicillin–streptomycin, 1% sodium pyruvate and puromycin 500 ng/ml at 37°C and 5% CO_2_.

### Plasmids

The DNA binding and dimerization domain of ZEBRA (residues 149–245) was cloned downstream of the tandem StrepII/FLAG-tag (54) to yield the plasmid p3928. The ZEBRA expression plasmid p509 is described elsewhere (55). All the plasmids encoding ZEBRA mutants were generated by introducing point mutations into plasmid p509. The luciferase plasmid p4376 was constructed by inserting a pentamer of a 24 bp long oligonucleotide (GGTGCTCATGAGCGAGGGCCAGAT, ZRE2 is underlined) into a basic luciferase reporter plasmid with a minimal EF1a promoter. The entire plasmid backbone of this reporter plasmid is free of CpGs (56). The plasmid p2670 is described elsewhere (57).

### DNA transfection

Transfection of DNA into HEK293 and ZEBRA knockout cells was performed using PEI max (Polysciences). During the preparation of the transfection mixture, cells were switched to Optimem minimal medium (Invitrogen). The DNAs were mixed with 0.3 ml (for six-well plate) or 6 ml (for 130 mm dish) Optimem and then 6 μl PEI (1 mg/ml in water) were added per μg DNA. The mixture was incubated for 15 min at room temperature and was added to the cells for 4–5 h. Then the transfection medium was replaced by standard medium.

For the protein extracts used in EMSAs, 1 × 10^7^ HEK293 cells per 130-mm dish were seeded the day before transfection. Each plate was transfected with 30 μg of plasmid DNA. For Western blot analysis, 8 × 10^5^ HEK293 cells were seeded into 6-well plates the day before transfection and 0.5 μg of plasmid DNA were transfected per well. For reporter assays, 8 × 10^5^ HEK293 cells were seeded into 6-well plates the day before transfection. Each well was cotransfected with 1 μg of reporter plasmid together with 5 ng of transactivator and 50 ng of DNA of a renilla-expressing plasmid as an internal control for data normalization. For EBV production, 8 × 10^5^ ZEBRA knockout cells were seeded into 6-well plates the day before transfection. Each well was transfected with 0.5 μg of ZEBRA expressing plasmid (p509 encoding wt ZEBRA or ZEBRA mutant derivatives based on p509), and 0.5 μg of p2670 plasmid DNA (57) and supernatants with EBV particles were harvested three days after DNA transfection.

### Electromobility shift assays

Electromobility shift assays (EMSAs) were performed with purified protein from HEK293 cells transiently transfected with Strep/FLAG:ZEBRA (p3928). Protein purification was performed as previously described (9). The oligos ZRE2for (ATAGCTTATGAGCGATTTTATC), meZRE2for (ATAGCTTATGAG^m^CGATTTTATC), ZRE2rev (ATGATAAAATCGCTCATAAGCT), meZRE2rev (ATGATAAAAT^m^CGCTCATAAGCT), ZREfor (ATAGCTTATGTGCAATTTTATC) and ZRErev (ATGATAAAATTGCACATAAGCT) containing the ZRE2 and ZRE5 from the BRLF1 promoter and the oriLyt, respectively, were used. EMSAs were performed as described previously (27).

### Protein lysates from transiently transfected 293T cells and western blot immunostaining

To compare the steady-state protein expression of ZEBRA and ZEBRA mutants, plasmid DNAs of expression plasmids encoding ZEBRA and its nine single amino acid mutants were chemically transfected into 293T cells using polyethyleneimine. Three days after DNA transfection the cells were collected, centrifuged and washed in cold PBS and were resuspended in RIPA lysis buffer (50 mM TRIS, 150 mM NaCl, 1% NP40, 0.5% DOC, 0.1% SDS, pH 8.0) complemented with protease and phosphatase inhibitors. Cell lysates were frozen at −80°C. After thawing on ice, the lysates were mixed and centrifuged at 13 000 rpm for 10 min at 4°C. Supernatants were collected and the protein amount was determined using the Pierce BCA Protein Assay (Thermo Scientific). Protein concentrations of the lysates were adjusted using RIPA lysis buffer (50 mM Tris, 150 mM NaCl, 1% NP40, 0.5% DOC, 0.1% SDS, pH 8.0). Lämmli buffer was added and identical protein amounts of the different samples (20 μg) were loaded on mini-Protean TGX Stain-free Precast gels from Biorad. After the runs, the gels were activated by a 45 s UV exposure and electroblotted onto nitrocellulose membranes. The membranes were blocked and incubated with the Z125 antibody (58) (1:100 of a raw hybridoma supernatant) overnight in TBS-T (25 mM Tris pH 7.4, 137 mM NaCl, 2.7 mM KCl, 0.1% Tween-20) with 5% (w/v) fat-free dry milk powder. The anti-mouse HRP (Cell signaling, #7076S) secondary antibody was used after dilution (1:10 000) in TBS-T to visualize the BZLF1 signals after adding ECL select Western Blotting Detection Reagent (Amersham). The membranes were scanned using the ChemiDoc Imaging sytem (Bio-Rad), and the images were analyzed and the signals quantitated after total cell protein normalization using the Image Lab 6.0.1 software (Bio-Rad).

### *In vitro* DNA methylation

CpG methylation *in vitro* was performed with the *de novo* methyltransferase M.SssI and S-adenosyl methionine as described (59).

### Luciferase reporter assays

Forty-eight hours post-transfection, the HEK293 cells were analyzed with the Dual-Luciferase Reporter Assay System (Promega). Luciferase activity was measured in a 96-well microplate luminometer (Orion II, Berthold).

### Quantitation of viral particles in cell supernatants

Three days post-transfection the cell supernatants of ZEBRA knockout cells were collected, filtered with 1.2 μm filters and kept at 4°C. The EBV genome contained in the producer cell line ZEBRA knockout carries the *egfp* gene, and infectious units are defined with the aid of Raji cells, which turn GFP-positive upon infection, allowing the direct assessment of the concentration of infectious EBV virions as green Raji units (GRU) per milliliter by flow cytometric analysis as described earlier (60,61).

## RESULTS

### Structure of the ZEBRA/meZRE2 complex

We crystallized ZEBRA’s DNA-binding domain in complex with a 19 base-pair (bp) DNA duplex containing the CpG-methylated ZRE2 site in the EBV promoter Rp (TGAG^m^CGA; hereafter meZRE2) and solved the structure at 2.5 Å resolution by molecular replacement (Supplementary Table S1). Unlike the single helix of a canonical bZIP domain, whose N- and C-terminal residues bind DNA and mediate coiled-coil dimer formation, respectively, ZEBRA’s C-terminal region folds back on and stabilizes an unusually short coiled coil (Figure 1B). Our crystal structure closely resembles previous ZEBRA structures bound to the AP-1 site (43) and to the Rp meZRE2 site in an alternate crystal form (42), apart from the dimerization domain which exhibits variable bending (Supplementary Figure S1a). This domain is implicated in diverse protein–protein interactions (62–67) and its flexibility may allow ZEBRA to adapt to different binding partners.

Each ZEBRA monomer recognizes one of the two meZRE2 half-sites, which we denote ‘A’ (half-site shared with AP-1) and ‘M’ (methylated half-site) (Figure 1B). Consequently, only a single (hereafter ‘CpG-proximal’) monomer senses the methylation state of meZRE2. In the previously reported ZEBRA/AP-1 structure, the two ZEBRA monomers interact symmetrically with the AP-1 site except with the central G^0^:C^0′^ base pair: residue Arg^190^ from one monomer makes base-specific contacts with the guanine whereas the same arginine from the other monomer interacts nonspecifically with the phosphate flanking the cytosine (43). The ZEBRA/meZRE2 complex preserves this asymmetry: the CpG-proximal Arg^190^ reads the G^0^ base while the CpG-distal arginine contacts the DNA backbone, with water-mediated H-bonds that bridge the A and M half-sites stabilizing this configuration (Figure 1E and Supplementary Figure S2).

ZEBRA recognizes the A half-site of meZRE2 essentially as in the complex with AP-1 (apart from a minor difference described in Supplementary Figure S1b). CpG-distal residues Asn^182^, Ser^186^ and Arg^190^ form direct or water-mediated H-bonds with the C^2′^, T^1′^ and C^0′^ bases, respectively, while seven basic residues (Arg^179^, Arg^183^, Arg^187^, Lys^188^, Arg^190^, Lys^192^ and Lys^194^) mediate electrostatic interactions with DNA phosphate groups (Figure 1E). The CpG-proximal monomer recognizes DNA bases in the M half-site through direct H-bonding interactions of Asn^182^ with G^2^, Ser^186^ with ^m^C^–2′^ and ^m^C^1^, and Arg^190^ with G^0^ and G^–1′^, while electrostatic interactions with the DNA backbone resemble those in the A half-site. Notably, ZEBRA makes more base-specific contacts with the M than with the A half-site, allowing CpG methylation to have a greater impact on specific site recognition.

### AP-1 and meZRE2 site geometry deviates at the CpG site

**Figure 2.**
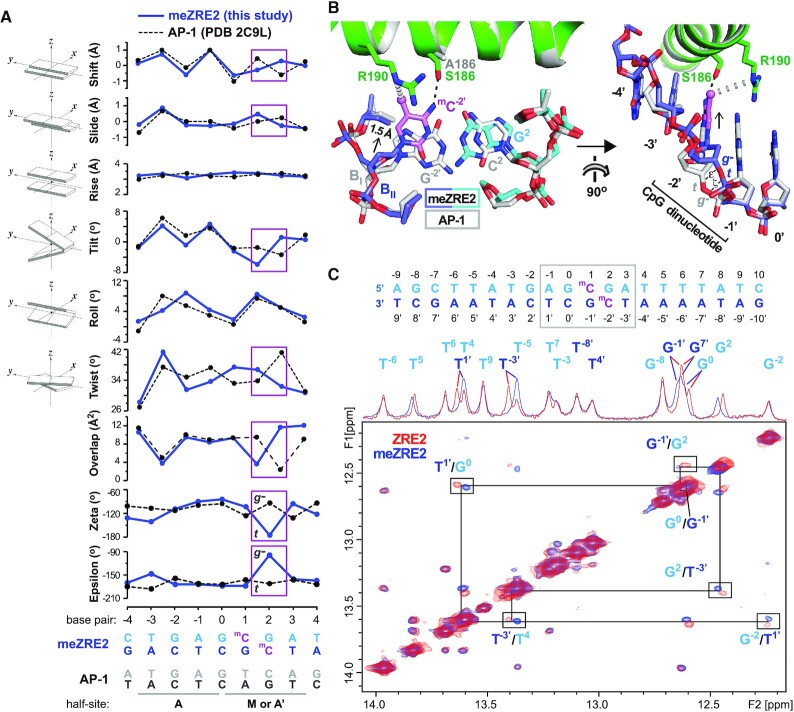
Deviations in AP-1 and meZRE2 site geometry localize to the methylation site. (**A**) Comparison of DNA geometry between the ZEBRA-bound AP-1 and meZRE2 sites. Base pair step parameters include translational (shift, slide, rise) and angular (tilt, roll, twist) parameters as well as the overlap between neighboring bases. Base pair numbering is that of the top (cyan) DNA strand. Base pair step parameters are plotted at the horizontal ordinate midway between the two base pairs comprising the step (e.g., values plotted at bp = 0.5 concern the base pair step G^0^C^0′^/^m^C^1^:G^–1′^). Large deviations at base pair steps 1/2 and 2/3 are boxed in violet. Parameter values were calculated using the program 3DNA (49). Images at the left of graphs are from (49). Backbone epsilon (ϵ) and zeta (ζ) angles are those of the bottom (blue) DNA strand. The B_II_ conformation is characterized by ϵ and ζ adopting a (*gauche^–^*, *trans*) [(*g^–^*, *t*)] configuration instead of the (*t*, *g^–^*) configuration that characterizes B_I_ (72,73). (**B**) Shift of meZRE2 nucleotide −2′ compared to AP-1. The structure of the ZEBRA/meZRE2 complex was aligned with that of the ZEBRA/AP-1 complex (PDB 2C9L). Black and gray dashed lines indicate H-bond and van der Waals interactions, respectively. The black arrow shows the direction of the shifted base. The B_I_ and B_II_ backbone conformations are indicated for nucleotide −2′. The B_II_ conformation allows the ^m^C^–2′^ base to interact with Ser^186^ and Arg^190^. The corresponding G^–2^ base of the AP-1 site would be unstable in this position because it cannot donate a H-bond to Ser^186^ (nor accept one since Ser^186^ already shares its hydroxyl proton with the closer T^1^ base). Right panel shows that the change in backbone geometry localizes to the linkage between nucleotides −1′ and −2′. Bonds related to torsion angles ϵ and ζ are indicated. (**C**) Imino region of 2D ^1^H, ^1^H-NOESY spectra and 1D spectra on top for ZRE2 (red) and meZRE2 (blue) DNA. The DNA sequences used and methylation sites are indicated on top. The sequential walk connecting the imino NMR signals in the central region (highlighted by a gray box in the DNA sequence) is indicated. For these base pairs notable chemical shift differences are observed upon methylation.

CpG methylation induces global changes in DNA structure (68–71), raising the possibility that ZEBRA’s enhanced affinity for methylated ZRE2 may reflect an altered DNA conformation. Comparing the ZEBRA-bound AP-1 and meZRE2 structures reveals nearly identical DNA geometry except at the CpG motif, where large differences are observed in base-step parameters involving the G^2^:^m^C^−2′^ base pair (Figure 2A). These differences primarily reflect a displacement of the ^m^C^–2′^ base towards the CpG-proximal ZEBRA monomer by 1.5 Å relative to the corresponding G^–2′^ base of AP-1 (Figure 2B), as previously observed (42). The displacement is made possible by the phosphate backbone adopting a B_II_ conformation instead of the more common B_I_ conformation of standard B-form DNA (72,73). B_II_ conformations can facilitate protein–DNA interactions by increasing the exposure of DNA bases in the major groove (74). In meZRE2, the B_II_ conformation allows the ^m^C^–2′^ to slip away and destack from the G^–1′^ base to form a H-bond with Ser^186^ and a van der Waals contact with Arg^190^ that stabilize the shifted base. A similar B_II_ conformation and base destacking was observed in the DNA-bound structure of the yeast transcription factor Ndt80 and may characterize several other structures in which an Arg residue interacts with a YpG dinucleotide motif (where Y is a pyrimidine nucleotide) (75,76). In the DNA-bound Ndt80 structure, where two TpG motifs are recognized by two Arg residues, a B_II_ conformation allows each 5′ T base to destack from the 3′ G and stack onto the guanidino group of the nearby Arg residue, which forms bidentate hydrogen bonds and is coplanar with the 3′ G base. Notably, whereas the Arg residues of Ndt80 and other YpG-recognizing proteins form cation–pi interactions with the destacked Y bases, in our ZEBRA structure the position of the Arg^190^ guanidino group relative to the ^m^C^−2′^ base ring is too far and too greatly off-centered to form a strong cation–pi interaction. The ^m^C base shift observed in our ZEBRA structure is also reminiscent of that previously described between methylated and unmethylated variants of the AP-1 site bound by homodimeric Jun (Supplementary Figure S3a) (42). However, whereas the shift in the ZEBRA complex is mediated by the B_I_→B_II_ transition of a single phosphodiester bond, that in the Jun complex is achieved through small backbone adjustments that extend over several nucleotides and widen the major groove (Supplementary Figure S3b). This contrast underscores the highly localized nature of the structural changes that differentiate the ZEBRA-bound meZRE2 and AP-1 sites.

We next used solution nuclear magnetic resonance (NMR) to assess potential conformational changes induced by CpG methylation of ZRE2. Homonuclear imino NOESY spectra were recorded for 20 bp duplexes containing methylated and unmethylated ZRE2 (Figure 2C). Imino chemical shifts were readily assigned and are consistent with B-DNA geometry. NOE connectivities for the imino walk are essentially identical for methylated and unmethylated ZRE2. However, imino signals within 2 base pairs of the CpG motif show significant chemical shift changes upon methylation, with the largest differences observed for crosspeaks involving the imino groups of G^–1′^ and G^2^ that base pair with the methylated cytosines. These differences are consistent with the change in electronic environment caused by the spatial proximity of the two methyl groups and may also reflect a small change in the DNA helical conformation for this region. The lack of more extensive spectral changes confirms that CpG methylation does not induce large-scale changes in ZRE2 conformation. Taken together, the NMR and crystallographic data indicate that ZEBRA’s selectivity for methylated over unmethylated ZRE2 does not involve changes in the overall DNA conformation but rather the recognition of structural features highly localized to the CpG site.

### CpG methylation marks are read by Ser^186^ and Arg^190^

ZEBRA binding to meZRE2 places the two CpG methylation marks in different stereochemical environments. ZEBRA recognizes the methyl groups of ^m^C^1^ and ^m^C^–2′^ through CpG-proximal residues Ser^186^ and Arg^190^, respectively (Figure 1C). Strikingly, the Ser^186^ side chain is positioned precisely on the local dyad axis that relates the two CpG methylation marks, allowing it to hydrogen bond with both ^m^C bases (Figure 1D). As previously observed (42), Ser^186^ senses the ^m^C^1^ methyl group through a van der Waals contact with its side chain methylene group. The *gauche*^+^ (*g*^+^) rotamer observed for this side chain would be weakly populated in unbound ZEBRA since serine has a high (∼85%) propensity to hydrogen bond with the helical backbone in the *g^–^* conformation (Supplementary Figure S4a) (77). In an unmethylated ZEBRA/ZRE2 complex, the *g*^–^ rotamer of Ser^186^ would compete with the *g*^+^ rotamer and attenuate DNA binding by reducing the number of base-specific H-bonds. By contrast, in the methylated complex the ^m^C^1^ methyl group sterically selects for the *g^+^* rotamer, thereby stabilizing the H-bonds with the ^m^C bases (Supplementary Figure S4b).

**Figure 3.**
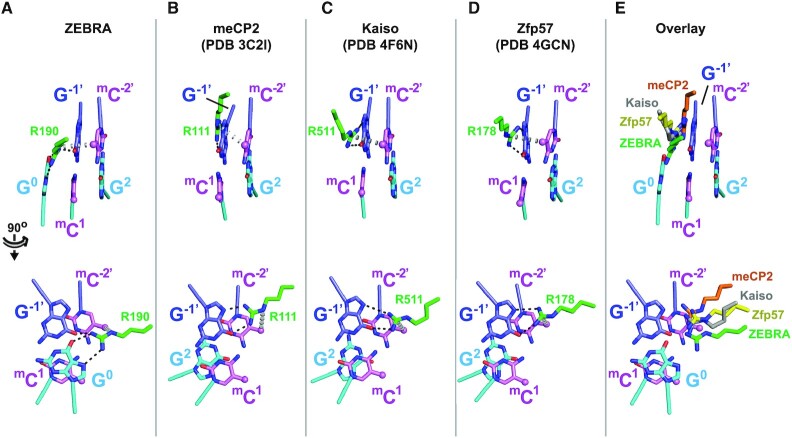
ZEBRA recognizes the ^m^C^–2′^ methyl group through an atypical ^m^C-Arg-G triad motif. (**A**) Recognition of the ^m^C^–2′^ methyl by ZEBRA residue Arg^190^. The top and bottom panels show views perpendicular to and along the DNA helical axis, respectively. Black and gray dashed lines indicate H-bonds and van der Waals contacts, respectively. (**B–D**) ^m^C-Arg-G triad observed in three methyl-CpG binding proteins showing that residues (B) Arg111 in meCP2 (79), (C) Arg511 in Kaiso (80) and (D) Arg178 in Zfp57 (81) adopt a similar orientation with respect to the methylated CpG motif. (**E**) Structural alignment of the ^m^C-Arg-G motifs from meCP2 (orange), Kaiso (gray) and Zfp57 (yellow) with the corresponding motif from ZEBRA. For simplicity only the DNA bases from ZEBRA are shown. ZEBRA’s Arg^190^ side chain forms bidentate H-bonds with G^0^ instead of with G^–1^ as observed in the canonical triad.

The methylation mark on ^m^C^–2′^ is sensed by Arg^190^ via its guanidino group (Figures 1C and 3A). This contact stabilizes the Arg^190^ side chain in a conformation that deviates slightly from that in the AP-1-bound structure (Supplementary Figure S1c). In the AP-1 complex, the Arg^190^ guanidino group forms bidentate H-bonds and is coplanar with the G^0^ base, whereas in the meZRE2 complex it twists out of this plane to form a bifurcated H-bond with the G^–1′^ base on the opposite strand. The resulting configuration is strikingly similar to the ^m^C-Arg-G triad observed in methyl-CpG binding proteins (MBPs), whereby a conserved arginine hydrogen bonds with the G base of the CpG motif and contacts the methyl group of the adjacent ^m^C base (analogous to G^–1′^ and ^m^C^–2′^ in our structure) (78–81) (Figure 3B–D). Compared to the canonical triad, ZEBRA’s Arg^190^ side chain is shifted, such that it forms only a bifurcated H-bond with G^–1′^ and instead forms bidentate H-bonds with G^0^ on the opposite DNA strand (Figure 3E). The importance of this configuration is underscored by the observation that swapping the central G^0^:C^0′^ base pair for a C:G markedly destabilizes the ZEBRA/meZRE2 complex (42) and by anti-ZEBRA ChIP-seq data showing that the central G:C base pair of ZEBRA-binding sites on viral and human genomic DNA is invariable (27,82).

### The two CpG methylation marks contribute unequally and independently to binding affinity

**Figure 4.**
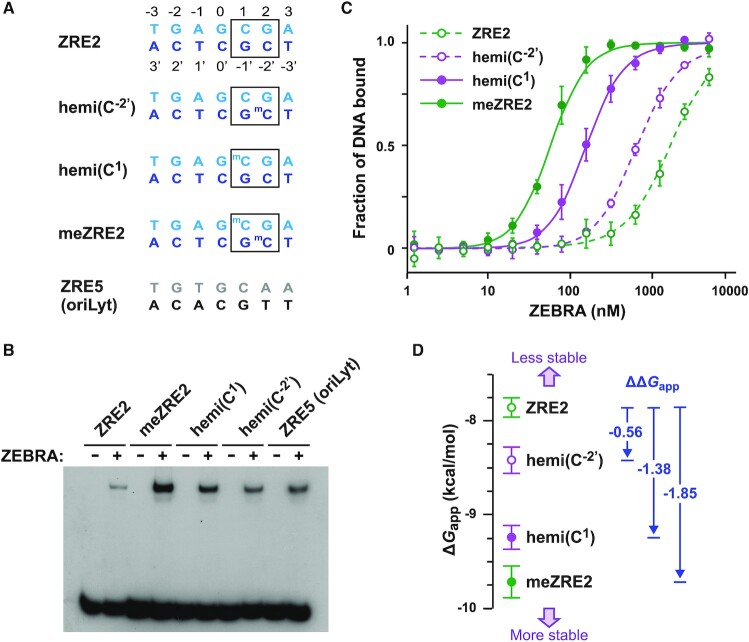
The two CpG methylation marks contribute unequally and independently to binding affinity. (**A**) ZRE sequences used for binding assays. Rp ZRE2 sequences (blue and cyan) were either unmethylated, hemi-methylated or symmetrically methylated as indicated. The AP-1-like sequence ZRE5 from oriLyt (black and gray) was included for comparison. (**B**) EMSA performed with affinity-purified Strep/FLAG:ZEBRA fusion protein transiently expressed in HEK293 cells. One representative experiment out of three is shown. (**C**) FP assays assessing the binding of ZEBRA to ZRE2 sites bearing 0, 1 or 2 methylation marks. (**D**) Apparent free energy of binding of ZEBRA/DNA complexes. Differences in the stability of complexes (ΔΔ*G*_app_ values) are indicated.

To determine the relative importance of the two CpG methylation marks for site recognition, we assessed ZEBRA’s ability to bind ZRE2 sites that were either unmethylated, fully methylated or hemi-methylated on C^1^ or C^–2′^ (Figure 4A). An electrophoretic mobility shift assay (EMSA) showed that ZEBRA bound both hemi-methylated sites more tightly than unmethylated ZRE2 but less tightly than fully methylated ZRE2 (Figure 4B), indicating that methylation on each DNA strand has an additive effect on binding affinity. ZEBRA bound the two hemi-methylated sites with similar affinity as a viral AP-1-like site (site ZRE5 from the lytic origin of replication), although binding appeared slightly stronger when hemi-methylation was on C^1^ compared to C^–2'^.

To better estimate ZEBRA’s affinity for differentially methylated ZRE2 sites we used a fluorescence polarization (FP) assay, in which the binding of ZEBRA to a fluorescently labeled DNA probe allows determination of the apparent dissociation constant (*K*_d,app_). Like the EMSAs, FP assays were performed in the presence of non-specific competitor DNA so as to emulate cellular conditions, where specific ZREs compete for ZEBRA against a large background of low-affinity binding sites in the genome. ZEBRA bound the fully methylated site with >20-fold higher affinity than the unmethylated ZRE2 (*K*_d,app_ of 75 nM versus 1.7 μM; Figure 4C and Table 1), in general agreement with previous findings (6,27,34,42). *K*_d,app_ values lower by a factor of ∼10 have been reported in the absence of competitor DNA (42). ZRE2 hemi-methylated on C^1^ reduced binding by a factor of 2.2 (*K*_d,app_ = 167 nM) compared to the fully methylated site. By contrast, hemi-methylation on C^–2′^ reduced binding by a factor of ∼9 (*K*_d,app_ = 670 nM), revealing that the two methylation marks contribute unequally to binding affinity, as suggested by the EMSA (Figure 4B).

Additional insights can be gained from the FP data by considering the apparent binding free energy, Δ*G*_app_ [calculated as -*RT*ln(1/*K*_d,app_) with temperature *T* and gas constant *R*] and how this quantity varies (ΔΔ*G*_app_) between different complexes. Plotting Δ*G*_app_ for the above ZEBRA/DNA complexes reveals that, compared to unmethylated ZRE2, the gain in complex stability obtained by fully methylating ZRE2 (ΔΔ*G*_app_ = −1.85 kcal/mol) closely matches the sum of the values obtained by hemi-methylating at C^1^ (−1.38 kcal/mol) and at C^–2′^ (−0.56 kcal/mol) (Figure 4D and Table 1). For comparison, the energy of a neutral H-bond in water is 0.5–1.5 kcal/mol, depending on the bond strength (83–85). Thus, each methyl group contributes independently to the enhanced affinity of ZEBRA for the fully methylated site, with the C^1^ and C^–2′^ methylation marks each providing approximately 75% and 25% of the binding energy, or roughly the equivalent of a strong and weak H-bond, respectively.

### Insights into the inhibitory effect of CpG hydroxymethylation

Besides CpG methylation, another epigenetic mark implicated in regulating EBV gene expression is cytosine 5-hydroxymethylation. This modification is mediated by ten-eleven translocation (TET) dioxygenases, which convert 5-methylcytosine (^m^C) to 5-hydroxymethylcytosine (^hm^C) (86). Loss of TET2 is implicated in the development of EBV-positive nasopharyngeal carcinoma (87) and may play a role in EBV-positive gastric carcinoma (88). In EBV-infected cells ZEBRA-mediated lytic reactivation is strongly reduced by ^hm^C modification of viral lytic promoters (87). *In vitro* studies have shown that ^hm^C modification markedly inhibits the binding of ZEBRA to CpG-containing ZREs relative to the methylated ZRE site (42,87). In agreement with these findings, FP assays showed that ZEBRA’s ability to bind meZRE2 was significantly compromised by hydroxymethylation (Supplementary Figure S5a,b). Binding was reduced to a similar degree (by a factor of ∼1.7) when either ^m^C^1^ or ^m^C^–2'^ was replaced by ^hm^C and further reduced (by a factor of 3.1) when both modifications were made, revealing that the two hydroxymethyl marks had an additive inhibitory effect. Interestingly, ZEBRA’s affinity for hydroxymethylated ZRE2 (hmZRE2) was comparable to that for the AP-1 site (Table 1), whose high abundance in the human genome might outcompete hmZRE2 for ZEBRA binding (see below).

To understand the inhibitory effect of hydroxymethylation, we modeled the structure of ZEBRA bound to a ^hm^C-modified ZRE2 site by replacing the two ^m^C nucleotides in our crystal structure by ^hm^C. A survey of high-resolution ^hm^C-containing DNA structures in the Protein Data Bank (PDB) revealed that the ^hm^C hydroxymethyl group preferentially adopts a *syn*-periplanar (*sp*) or (+)-clinal (+*c*) conformation (Supplementary Figure S5c,d). This rotational dimorphism is favoured by direct or water-mediated H-bonds with specific atoms of the CpG dinucleotide (89) and is consistent with energy calculations (90). Our structural model predicts that the *sp* and +*c* conformations of ^hm^C^1^ would give a strong steric clash with the backbone atoms of Arg^183^ and Asn^182^, respectively (Supplementary Figure S5e), while the +*c* conformation of ^hm^C^–2′^ would clash with the guanidino group of Arg^190^ that interacts with the G^0^ and G^1′^ bases (Supplementary Figure S5f). Relieving these clashes would require an increased separation between the protein backbone and DNA bases that would disrupt the H-bonds between Ser^186^ and the C^1^ and C^–2′^ bases (Figure 1D) and thereby destabilize the complex.

### Ser^186^ and Arg^190^ are key determinants of high-affinity meZRE2 binding

**Figure 5.**
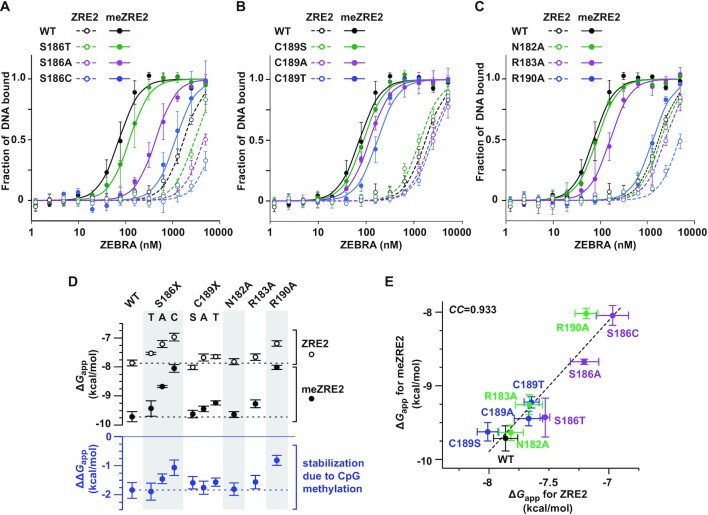
Effect of ZEBRA point mutations on binding affinity and selectivity for methylated ZRE2. (**A–C**) FP assays assessing the effect of (A) Ser^186^ mutants, (B) Cys^189^ mutants and (C) alanine substitutions of Asn^182^, Arg^183^ and Arg^190^ on ZEBRA’s ability to bind methylated and unmethylated ZRE2. The drop in affinity observed for the S186T mutant is consistent with a steric clash predicted between Arg^190^ and the threonine methyl group that would hinder optimal positioning of the threonine hydroxyl group relative to the two ^m^C bases. The drop in affinity observed for the C189A mutant is consistent with the loss of non-specific interactions between the Cys^189^ thiol and the T^–3^ and T^–3′^ phosphate groups. Modeling suggests that the decreased affinity observed for the C189T mutant is due to steric constraints involving the threonine and T^–3′^ methyl groups that result in suboptimal H-bonding geometry. Effects observed for the remaining mutants are discussed in the main text. (**D**) Plot of apparent free binding energies (Δ*G*_app_) of ZEBRA/DNA complexes derived from *K*_d, app_ values determined in (A–C). The difference in stability between ZRE2 and meZRE2 complexes (ΔΔ*G*_me/Z_) is indicated below. (**E**) Plot of Δ*G*_app_ values comparing the stability of meZRE2 and ZRE2 complexes; CC, correlation coefficient.

To evaluate the significance of protein contacts with the ^m^CpG motif observed in our crystal structure, we examined the effect of single point mutations on ZEBRA’s ability to bind meZRE2 in FP assays (Table 1). We first mutated Ser^186^ to either a threonine, alanine or cysteine. Whereas threonine replacement gave a modest drop in affinity (by a factor of 1.6) consistent with stereochemical considerations (detailed in Figure 5 legend), replacement by alanine caused a more pronounced reduction in affinity (by a factor of 6; Figure 5A), in line with previous findings (34,40–42) and consistent with the loss of three H-bonds that Ser^186^ makes with the M and A half-sites (Figure 1C–E). A more dramatic decrease in affinity (by a factor of >16) was observed when Ser^186^ was replaced by cysteine. This is surprising given the nearly isosteric cysteine and serine side chains and contrasts with the inverse serine substitution of Cys^189^, which had virtually no effect (Figure 5B). We surmised that disulfide crosslink formation might explain the poor activity of the S186C mutant (Supplementary Figure S6a); however, experiments do not support this hypothesis (Supplementary Figure S6b–d). Interestingly, a dramatic loss of binding activity was also reported for a bacterial sulfate-binding protein when a Ser residue that donates a hydrogen bond to the sulfate ligand was substituted by Cys, compared to a much weaker effect when Ala or Gly was substituted (91). This loss of activity was attributed to differences in the size and preferred angles of the Cys thiol group relative to the Ser hydroxyl group and to the differential work required to polarize these groups (91,92). Similar effects might explain the poor binding activity of the ZEBRA S186C mutant. In contrast to serine, replacing Cys^189^ by an alanine or threonine decreased binding affinity for meZRE2 by a factor of 1.6 or 2.3, respectively (Figure 5B). These results can be rationalized structurally (Figure 5 legend) and are consistent with a recent protein binding microarray study that found reduced meZRE2 binding for ZEBRA mutants C189A and C189T (93).

Finally, we investigated the effect of alanine substitutions at positions 182, 183 and 190 (Figure 5C). Asn^182^ is conserved across canonical bZIP proteins and is important for specific AP-1 site recognition, with mutation to alanine resulting in the loss of high-affinity binding (94). By contrast, alanine substitution of Asn^182^ had little effect on ZEBRA’s affinity for meZRE2 (Figure 5C and Table 1). This difference is partly due to the fact that, whereas the conserved Asn of bZIP proteins forms four H-bonds with the AP-1 site (95,96), ZEBRA residue Asn^182^ forms only two with meZRE2, of which one is weak (Figure 1C,E and Supplementary Figure S1b), and might also reflect a tighter protein–DNA interface enabled by the smaller alanine side chain that compensates for the disrupted H-bonds. Alanine substitution of Arg^183^ had only a small effect on binding affinity, consistent with this residue’s non-specific interaction with the phosphate backbone. In contrast, alanine substitution of Arg^190^ caused a drastic loss of binding affinity (by a factor of 17), consistent with the loss of multiple specific and non-specific interactions that this residue mediates with the M and A half-sites, respectively (Figure 3A and Supplementary Figure S2).

In summary, of the five alanine substitutions tested, mutants S186A and R190A greatly reduced ZEBRA’s affinity for meZRE2, whereas mutants N182A, R183A and C189A had at most only a modest effect on binding. Thus, the same residues that sense the CpG methylation status within meZRE2 are also critically required for high-affinity binding.

### Selectivity for meZRE2 is robust and mostly independent of base-specific contacts

In parallel to the above experiments we also assessed point mutants for their ability to bind the unmethylated ZRE2 site. In general, mutations affected the binding of ZRE2 similarly to that of meZRE2. For example, the S186T mutation reduced the affinity for meZRE2 and ZRE2 by factors of 1.6 and 1.7, corresponding to a loss in complex stability of 0.28 and 0.33 kcal/mol, respectively (Figure 5A and D). Indeed, the results for the nine mutants revealed that the Δ*G*_app_ values for the methylated and unmethylated complexes correlated strongly (Figure 5E and Table 1).

Interestingly, for certain mutations the impact on binding affinity differed significantly between ZRE2 and meZRE2. In particular, the S186A mutation decreased the binding affinity for ZRE2 by a factor of 3, compared to a factor of 6 for meZRE2, implying a 50% drop in selectivity for the methylated site. This is consistent with CpG methylation stabilizing Ser^186^ in a *g^+^* conformation that mediates two H-bonds with the DNA (Supplementary Figure S4b), which are eliminated by the alanine substitution. In the unmethylated complex the reduced prevalence of the *g^+^* rotamer decreases the effective number of H-bonds disrupted by the mutation, explaining the weaker destabilizing effect. More strikingly, the R190A mutation reduced the binding affinity for meZRE2 by a factor of 17 but that for ZRE2 by only a factor of 3, hence reducing the selectivity for the methylated site by a factor of 5.5. This implies that CpG methylation has a much weaker stabilizing effect on the mutant ZEBRA/DNA complex compared to the WT (ΔΔ*G*_app_ increased by >1 kcal/mol; Figure 5D and Table 1). This finding is consistent with the ^m^C-Arg-G triad geometry in which the ^m^C^−2′^ methyl group stabilizes Arg^190^ in a conformation that hydrogen bonds with the G^0^ and G^–1′^ bases (Figure 3). Truncation of this side chain renders the R190A mutant unable to sense the methylation status of the C^–2′^ base, explaining why CpG methylation more weakly stabilizes the mutant complex.

Notably, no mutations were identified that abolished selectivity for methylated ZRE2 (even the R190A mutant retained 4-fold selectivity). In particular, selectivity did not require ZEBRA-specific residue Ser^186^, since the alanine mutant still discriminated efficiently (12-fold) in favor of meZRE2. Taken together these observations reveal that ZEBRA’s selectivity for methylated DNA is remarkably robust and only weakly depends on the integrity of individual base-specific contacts.

### Ser^186^ confers binding selectivity for meZRE2 over AP-1

**Figure 6.**
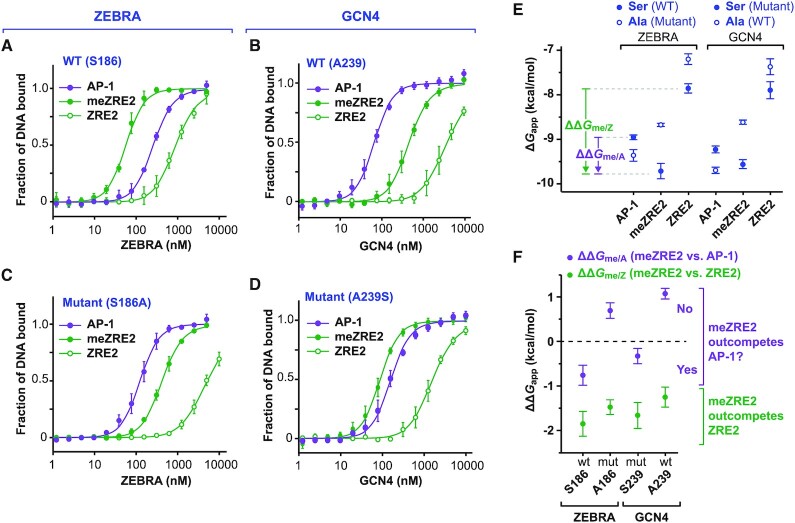
Ser^186^ confers binding selectivity for meZRE2 over AP-1. (**A–D**) FP assays assessing the relative binding affinity of (A) WT ZEBRA, (B) WT GCN4, (C) ZEBRA mutant S186A and (D) GCN4 mutant A239S for the AP-1, ZRE2 and meZRE2 sites. (**E**) Plot of apparent free binding energies (Δ*G*_app_) of protein/DNA complexes derived from *K*_d, app_ values determined in (A–D). The quantities ΔΔ*G*_me/Z_ and ΔΔ*G*_me/A_ are illustrated for WT ZEBRA. (**F**) Values of ΔΔ*G*_me/Z_ and ΔΔ*G*_me/A_ plotted for the indicated ZEBRA or GCN4 protein illustrating the stability of the meZRE2-bound protein relative to that of the ZRE2 (ΔΔ*G*_me/Z_) or AP-1 (ΔΔ*G*_me/A_) complex.

Since ZEBRA is known to recognize two (AP-1-like and CpG-containing) classes of ZREs, we examined its affinity for methylated and unmethylated ZRE2 relative to that for the AP-1 site. As reported above, ZEBRA binds meZRE2 with >20-fold selectivity over ZRE2 (*K*_d,app_ values of 75 and 1700 nM, respectively). FP assays showed that ZEBRA bound the AP-1 site with an affinity intermediate between these two values (*K*_d,app_ of 270 nM; Figure 6A and Table 1). Consistent with this observation, the EMSA in Figure 4B shows that ZEBRA’s affinity for another AP-1-like site, oriLyt ZRE5, is also intermediate between that for ZRE2 and meZRE2. For comparison, we examined the site selectivity of GCN4, a canonical bZIP protein from yeast that recognizes AP-1 sites (95). As expected, GCN4 bound AP-1 tightly (*K*_d,app_ of 78 nM) with 50-fold selectivity over ZRE2 (Figure 6B). GCN4 bound meZRE2 with an intermediate affinity (*K*_d,app_ of 480 nM) that was 8 times stronger compared to ZRE2 and 6 times weaker compared to AP-1. Thus, whereas the binding selectivity of ZEBRA follows the order meZRE2 > AP-1 > ZRE2, that of GCN4 switches the order of AP-1 and meZRE2. This difference arises because ZEBRA’s affinity is both lower for AP-1 and higher for meZRE2 compared to GCN4.

Like most bZIP proteins, GCN4 has an alanine (Ala^239^) instead of ZEBRA’s Ser^186^ residue. Mutating the alanine residue in GCN4 to serine reduced its affinity for the AP-1 site by a factor of 2 and enhanced that for ZRE2 and meZRE2 site 2.5- and 5-fold, respectively, resulting in ZEBRA-like selectivity (meZRE2 > AP-1 > ZRE2) (Figure 6D). The inverse mutation on ZEBRA (S186A) had the opposite effect: the affinity for AP-1 increased 2-fold, while that for ZRE2 and meZRE2 decreased by factors of approximately 3 and 6, respectively, yielding GCN4-like selectivity (AP-1 > meZRE2 > ZRE2; Figure 6C). Comparing the stabilities (Δ*G*_app_ values) of all 12 protein/DNA combinations reveals a striking similarity between corresponding ZEBRA and GCN4 complexes (Figure 6E). Whereas the serine-containing ZEBRA and GCN4 variants form the most stable complexes with meZRE2, the alanine substitution stabilizes the AP-1 complex (downward shift of Δ*G*_app_) while destabilizing both the meZRE2 and ZRE2 complexes (upward shifts).

The above trends become evident when the differences in complex stability are expressed as ΔΔ*G*_app_ values. For convenience, we denote ZEBRA’s ability to discriminate meZRE2 from either ZRE2 or AP-1 as selectivity of type ‘me/Z’ or ‘me/A’ (defined as the ratio of ZEBRA’s apparent binding affinity, 1/*K*_d,app_, for meZRE2 to its apparent affinity for ZRE2 or AP-1, respectively; Figure 1A) and denote the corresponding differences in apparent binding free energy as ΔΔ*G*_me/Z_ or ΔΔ*G*_me/A_, respectively. The latter quantities are illustrated for WT ZEBRA in Figure 6E and plotted for the four ZEBRA and GCN4 proteins in Figure 6F (large negative ΔΔ*G* values correspond to high positive selectivity). Methylating ZRE2 induces a similar stabilization of DNA-bound ZEBRA (ΔΔ*G*_me/Z_ = −1.85 kcal/mol) and GCN4 (−1.66 kcal/mol) when residue 186 or 239 is serine. An alanine at this position yields a small increase (∼0.4 kcal/mol) in ΔΔ*G*_me/Z_, which nevertheless remains below −1.2 kcal/mol for the WT and mutant forms of both proteins, reflecting their shared high selectivity for meZRE2 over ZRE2. In contrast, the Ser→Ala substitution induces a large shift (+1.4 kcal/mol) in ΔΔ*G*_me/A_, which flips from a negative to a positive value for both proteins. This inversion of sign corresponds to the switch in binding-site ranking described above: whereas meZRE2 outcompetes the AP-1 site for the serine-containing ZEBRA and GCN4 variants (ΔΔ*G*_me/A_ < 0), AP-1 outcompetes meZRE2 for the alanine variants (ΔΔ*G*_me/A_ > 0). Thus, the identity of the residue at or equivalent to position 186 determines which of the two binding sites these bZIP proteins preferentially bind.

### Transactivation of a CpG-methylated promoter mirrors meZRE2 binding affinity

**Figure 7.**
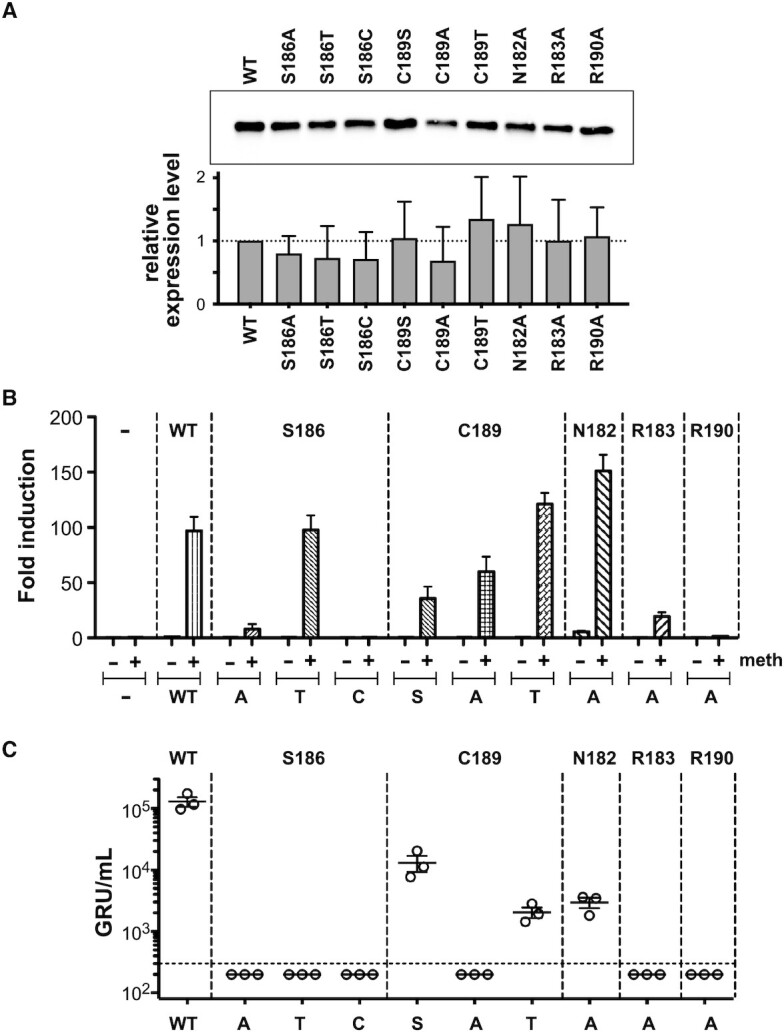
Effect of ZEBRA point mutations on promoter activation and viral production. (**A**) Quantitation of steady state protein levels of WT and mutant ZEBRA proteins. Top: Representative image of ZEBRA proteins after western blot immunodetection. Bottom: Statistical analysis based on six independent biological replicates and western blot analyses. Cellular total protein levels were quantified after membrane blotting and used to normalize the levels of the ZEBRA protein signals after immunostaining. WT ZEBRA signal levels were set to 1.0, and the relative expression of the ZEBRA mutants were calculated after protein normalization. 293T cells were transfected with the WT ZEBRA expression plasmid and nine plasmids encoding the ZEBRA mutants as indicated. Three days post-infection, the cells were lysed, protein lysate concentrations were determined and equal protein amounts were subjected to quantitative western blot analysis using the western blot stain-free TGX Biorad Normalization approach (Bio-Rad). Blots were probed with the Z125 monoclonal antibody (58). Mean and standard deviation are shown. (**B**) Luciferase reporter assays assessing the ability of different ZEBRA mutants to activate a ZRE- or meZRE-containing promoter. Unmethylated and fully CpG-methylated reporter constructs were analyzed in the presence or absence of the indicated ZEBRA expression plasmid. After data normalization to a luciferase control plasmid free of promoter elements, the *x*-fold differences were calculated. Each experiment was performed three times and the means and standard deviations are depicted. (**C**) All ZEBRA mutants are impaired to different degrees in their capacity to reactivate virus production *in vitro*. ZEBRA knockout cells were transfected with the plasmids expressing WT ZEBRA or the indicated mutants. Viral titers in the cell supernatants were analyzed by infecting Raji cells and are provided as ‘green Raji units’ (GRU) per ml. Results from three independent experiments, including the means and standard deviations, are shown. The horizontal dotted line represents the detection limit of our assay.

We next assessed the ability of ZEBRA mutants to transactivate a CpG-methylated promoter in a luciferase reporter assay. Pentamers of the CpG-containing ZRE site from the EBV BSLF2/BMLF1 promoter were inserted into a luciferase reporter plasmid that was otherwise devoid of CpG motifs (27,56). Following mock treatment or treatment with a *de novo* methyl transferase to introduce CpG methylation of the five ZREs, the plasmid DNA was transiently transfected into HEK293 cells together with an expression plasmid encoding WT or mutant ZEBRA protein. Quantitative western blot analysis showed that mutants were expressed at the expected size and at near WT levels (Figure 7A). As expected, transfection with the WT ZEBRA protein led to strong transactivation of the methylated promoter (97-fold higher relative to a luciferase control plasmid free of promoter elements) but yielded only background activation of the unmethylated promoter (Figure 7B). Compared to WT, the transactivation of the methylated promoter was similar or higher for three mutants (S186T, C189T and N182A), was reduced by factors of 2 to 5 for three other mutants (C189A, C189S, R183A) and was reduced by a factor of >10 for the three remaining mutants (S186A, S186C and R190A). Interestingly, the level of transcriptional activation closely mirrored the *in vitro* binding stability measured for ZEBRA mutants in complex with meZRE2 (Supplementary Figure S7a). Thus, the degree of transcriptional activation in this assay showed a gradual response commensurate with ZEBRA’s affinity for the meZRE2 site.

### Viral lytic activation by ZEBRA mutants suggests a threshold-like response

Next, we tested whether our ZEBRA mutants were able to induce EBV’s lytic cycle. We used a HEK293 cell line stably transfected with an EBV genome encoding green fluorescent protein (GFP) and unable to express ZEBRA. Transient transfection of these cells with ZEBRA and BALF4 (encoding the viral envelope glycoprotein gp110) leads to the production of viral particles, whose concentration is assessed by infecting (and hence inducing GFP expression in) Raji cells, followed by flow cytometry to quantify the green Raji units (GRU) per ml, as previously described (61). As expected, a strong viral lytic response was observed following transfection with the WT construct (Figure 7C). In contrast, we failed to detect viral particles in the culture supernatant following transfection with six of the ZEBRA mutants (R183A, C189A, R190A and all three S186 mutants), indicating that these mutations abrogate ZEBRA’s ability to activate the lytic cycle. The remaining three mutants (C189A, C189T and N182A) were able to induce viral production, although less efficiently than WT ZEBRA, reducing viral production (GRU/ml) by a factor of 10 to 50.

**Figure 8.**
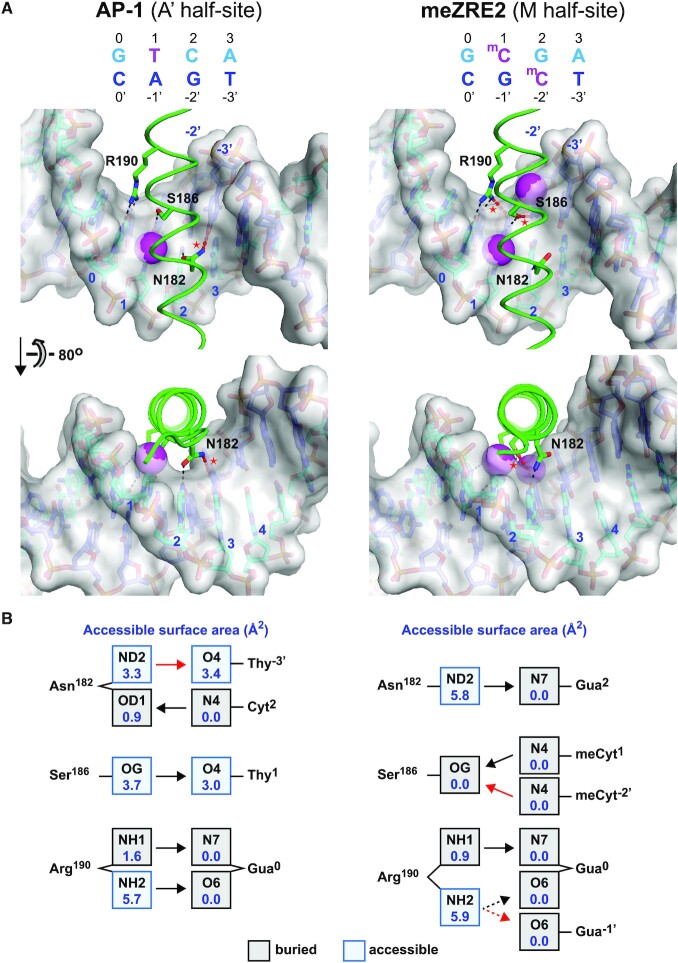
H-bond interactions with the unique (A’ and M) half-sites of AP-1 and meZRE2. (**A**) Base-specific hydrogen bond interactions mediated by residues Asn^182^, Ser^186^ and Arg^190^ and nucleotide bases in the A' half-site of AP-1 (left) and corresponding M half-site of meZRE2 (right). The structures of the AP-1 and meZRE2 complexes are from PDB 2C9L and this study, respectively. Because PDB 2C9L contains the S186A mutation, the Ser^186^ side chain conformation for the A' half-site was taken from the A half-site of the ZEBRA/meZRE2 structure. Hydrogen bonds unique to each complex are shown in red and marked by a red asterisk. The unique H-bond in the AP-1 complex between Asn^182^ and T^–3^ is accessible to solvent at the periphery of the protein/DNA interface whereas the unique H-bond in the meZRE2 complex between Ser^186^ and ^m^C^–2′^ is buried in the center of the interface. Methyl groups on AP-1 base T^1^ and meZRE2 bases ^m^C^1^ and ^m^C^–2′^ are shown as magenta spheres, highlighting that only the ^m^C^–2′^ methyl group is unique to meZRE2. (**B**) Accessible surface areas (ASAs) of H-bond donor and acceptor atoms. ASAs (shown in blue) were calculated using the program Areaimol of the CCP4 suite (46). Buried and solvent-accessible atoms are shown in gray and light blue squares, respectively. Arrows indicate H-bond directionality. Arrows in red correspond to H-bonds shown in red in panel (A). The dashed arrows indicate a bifurcated H-bond. The number of H-bonds is identical for the two half-sites if one considers that the bifurcated H-bond mediated by Arg^190^ (a three-centered interaction in which a single proton is shared between two acceptor atoms) is comparable in strength to a canonical H-bond (101,102).

The above data correlate poorly with the ability of mutants to activate transcription in the luciferase reporter assay (Supplementary Figure S7b), reflecting the non-linear dependence of viral production on meZRE2-containing promoter activation. This is not surprising since EBV lytic activation is probably regulated by several genes whose expression depends on ZEBRA binding to various ZRE and meZRE sites. ZEBRA is also an essential replication factor that needs to bind the lytic origin of DNA replication to promote efficient viral DNA amplification (97,98). Interestingly, the ability of ZEBRA mutants to activate the lytic cycle was strongly associated with their ability to bind meZRE2 with a *K*_d,app_ below ∼100 nM (Δ*G*_app_ < −9.5 kcal/mol) in our FP assays (the only exception being mutant C189T, which activated lytic replication with a *K*_d,app_ of 170 nM; Supplementary Figure S7c). This suggests that lytic activation involves a threshold-like response to ZEBRA/meZRE2 complex formation. This hypothesis has been recently confirmed in a model that allows a dose-dependent evaluation of ZEBRA’s ability to induce the lytic phase of EBV (see Figure 8 in (99)).

## DISCUSSION

In this study we investigated ZEBRA’s interactions with its two classes of ZRE target sites. We show that ZEBRA residues Ser^186^ and Arg^190^ play a key role both in establishing high-affinity binding with meZRE2 and in recognizing its CpG methylation status. Methyl-CpG recognition is facilitated by a DNA backbone conformation that allows the ^m^CpG motif to form an extra H-bond with Ser^186^ and to contact Arg^190^ through a non-canonical ^m^C-Arg-G triad (Figures 2B and 3A). Binding assays with hemi-methylated DNA revealed that the CpG methylation mark read by Ser^186^ enhances ZEBRA’s affinity for meZRE2 to a 3-fold greater extent than that read by Arg^190^ (Figure 4). Conversely, alanine point mutations revealed that the R190A mutant had a 3-fold lower meZRE2-binding affinity than the S186A mutant (Figure 5A,C and Table 1). Thus, while both residues participate in high-affinity binding and ^m^CpG recognition, they make unequal and opposite contributions to these activities. Remarkably, all individual ZEBRA point mutants investigated retained a strong binding preference for meZRE2 over ZRE2 (Figure 5 and Table 1), suggesting that an inherent difference in the physicochemical behaviour of these two sites might contribute significantly to such selectivity (denoted ‘me/Z’ selectivity). Indeed, computational studies have shown that methylation preferentially drives a CpG-containing DNA duplex into a protein-DNA complex compared to the unmethylated duplex (100). Consistent with this idea, the mutation that most severely compromises me/Z selectivity, R190A, is predicted to induce a large increase in methylcytosine solvent accessibility within the ZEBRA/meZRE2 complex, whereas the less compromising S186A mutation is predicted to cause only a marginal increase (Supplementary Figure S8).

ZEBRA binds more tightly to meZRE2 than to the consensus AP-1 site (Figure 6A) or to an AP-1-like site (Figure 4B). In agreement with this observation, a recent genome-wide study found that, at low levels of intracellular expression, ZEBRA predominantly associates with CpG-containing ZRE motifs and when expressed at higher levels additionally associates with AP-1-like sequences (99). What is the molecular basis for this type of selectivity (denoted ‘me/A’ selectivity)? Because AP-1 and meZRE2 share the A half-site in common, the answer lies in how ZEBRA interacts differently with the unique M and A' half-sites (Figure 8A). Surprisingly, these two interfaces have the same number of H-bonds. Except for Asn^182^ and Ser^186^, all DNA-contacting residues mediate similar interactions in the two complexes. Asn^182^ makes two H-bonds with the A' half-site (with C^2^ and T^–3′^) but only one with the M half-site (with G^2^). Conversely, Ser^186^ makes only one H-bond with the A' half-site (with T^1^) but two with the M half-site (with ^m^C^1^ and ^m^C^–2′^), and so the total H-bond count is identical for both complexes [considering single and bifurcated H-bonds as equivalent (101,102); see Figure 8B legend]. Importantly, however, these H-bonds are located in different environments: the extra H-bond (between Asn^182^ and T^–3′^) in the AP-1 complex is exposed to solvent, whereas the extra H-bond (between Ser^186^ and ^m^C^–2′^) in the meZRE2 complex is buried (Figure 8A,B). Buried H-bonds are more stable (by up to 1.2 kcal/mol (103)) than those accessible to solvent since water competes for the H-bond donor and acceptor sites (104–106). The extra methyl group on ^m^C^–2′^ also makes the M half-site more hydrophobic than the A' half-site, and so burying the larger hydrophobic surface would yield a greater entropic gain for the meZRE2 complex. Taken together, enhanced H-bond stability and a larger hydrophobic effect could reasonably account for the different stabilities of the meZRE2 and AP-1 complexes.

Unlike ZEBRA, the canonical bZIP protein GCN4 binds the AP-1 site more tightly than meZRE2 (Figure 6B). Remarkably, swapping ZEBRA’s Ser^186^ for an alanine and the corresponding GCN4 Ala^239^ for a serine inverted the me/A selectivity of both proteins (Figure 6C–F). These findings agree with previous studies that reported enhanced affinity of the ZEBRA S186A mutant for the AP-1 site (39,41,107), decreased affinity of the same mutant for the meZRE2 site (34,35,42) and enhanced affinity of Fos and Jun for methylated ZRE sites when the corresponding Ala→Ser mutations were made (41,42). The fact that the S186A mutation only modestly reduces me/Z selectivity but dramatically inverts me/A selectivity suggests that the inability of this mutant to induce lytic gene expression and disrupt viral latency is not due to its poorer discrimination of methylated and unmethylated CpG motifs, but rather its sequestration by AP-1 sites preventing recruitment to meZREs. Since transcription factor sequestration by competing DNA binding sites can lead to a threshold-like dose-response of their target promoters (108–110), this may explain the threshold-like behaviour we observe for ZEBRA mutants in our viral lytic activation assays.

The above findings suggest a novel interaction model for understanding ZEBRA’s dual transactivating functions during EBV infection that integrates both me/Z and me/A selectivity (Supplementary Figure S9). During prelatency when the incoming genomic EBV DNA is still unmethylated, ZEBRA has low affinity for the unmethylated CpG-containing ZRE sites in lytic viral promoters and preferentially binds methylated CpG-containing cellular ZREs and AP-1 sites, thereby activating genes that promote B cell proliferation and help establish latency (Supplementary Figure S9a, left). Following extensive methylation of the latent viral genome as early as two to three weeks after infection (18), CpG-containing viral ZREs surpass AP-1 sites in their binding affinity for ZEBRA, allowing ZEBRA to activate viral lytic gene expression upon its induced expression at the onset of EBV’s lytic phase (Supplementary Figure S9a, right). The ZEBRA S186A mutant fails to activate lytic expression because the decreased affinity for meZRE2 results in sequestering of the mutant protein by competing DNA sequences—including specifically AP-1 sites, which are highly abundant in the human genome (111,112) and whose affinity for ZEBRA is enhanced by the mutation (Supplementary Figure S9b). Indeed, a genome-wide ChIP-seq analysis identified >5 × 10^5^ AP-1-like sites bound by ZEBRA when ZEBRA expression was induced in Raji cells (99). Similarly, although cellular AP-1 proteins such as Fos and Jun preferentially bind meZRE2 over ZRE2 sites, they fail to activate viral lytic genes because their affinity for AP-1 sites exceeds that for meZRE2 (Supplementary Figure S9b). This selectivity is inverted by the Ala→Ser mutations at positions equivalent to ZEBRA Ser^186^, allowing these proteins to overcome AP-1 site sequestration and activate lytic gene expression (41,113) (Supplementary Figure S9a).

The hydroxyl group at residue 186 that endows ZEBRA with me/A selectivity is strikingly parsimonious from not only a structural but also a molecular evolutionary perspective. Of the six possible serine codons, a TCC codon specifies Ser^186^ whereas a GCC codon specifies the corresponding alanine in several of ZEBRA’s closest human orthologs (Supplementary Figure S10), suggesting that me/A selectivity may have arisen through a single G→T transition. Given that the most frequent substitution mutation of alanine is to serine (114), bZIP proteins would seem poised to evolve me/A selectivity. The fact that most have conserved the alanine indicates a strong selection pressure against accepting a mutation here. Indeed, an alignment of human bZIP proteins reveals only two exceptions where the alanine is not conserved (Supplementary Figure S11). The first is CREB3 regulatory factor (CREBRF). Like ZEBRA, CREBRF has a TCC-encoded serine (Supplementary Figures S10b and S11) and its ortholog in drosophila associates with CpG-containing motifs (115), raising the possibility that CREBRF may preferentially bind methylated CpG-containing sites, as detailed in the legend of Supplementary Figure S11.

The second exception comprises the CCAAT/enhancer-binding protein (C/EBP) family of bZIP proteins, which have a valine corresponding to Ser^186^. These proteins recognize the C/EBP site (TTGCGCAA), which is bound with enhanced affinity by C/EBPβ when methylated on the central CpG motif (116). C/EBP proteins preferentially recognize the C/EBP site over alternate sequences such as the c/AMP response element (CRE), an 8-bp motif comprising two AP-1 A' half-sites (TGACGTCA, closely resembling the 7-bp AP-1 site TGAGTCA). Mutating the unique valine to alanine greatly enhances the affinity of C/EBPα for the CRE site (117), analogous to how the S186A mutation enhances ZEBRA’s affinity for the AP-1 site. Moreover, C/EBPα is sequestered to pericentromeric heterochromatin by ‘natural decoy’ C/EBP consensus sites located within tandem α-satellite DNA repeats. The Val→Ala mutant reduces sequestration by these decoy sites and permits binding to functional target sites, enhancing the transcriptional output from c/EBPα-responsive promoters (108). Thus, like ZEBRA, C/EBP proteins exhibit two types of site selectivity, one between different methylation states of the same site and the other between two classes of response element, with the latter selectivity altered by mutating the unique valine to alanine.

In conclusion, ZEBRA has hitherto been viewed as an unusual bZIP protein because it could preferentially bind and activate methylated promoters thanks to its unique Ser^186^ residue. However, our findings reveal that the functional significance of Ser^186^ is not that it enables ZEBRA to selectively bind methylated over unmethylated CpG-containing ZREs—an activity shared with other bZIP proteins—but that it enables ZEBRA to bind methylated ZREs preferentially over AP-1 sites. Knowledge of ZEBRA’s two types of site selectivity clarifies our understanding of the competing molecular interactions that govern ZEBRA-dependent gene expression and should facilitate future studies aimed at unravelling ZEBRA’s diverse roles in EBV infection and EBV-associated diseases.

## DATA AVAILABILITY

Atomic coordinates and structure factors for the reported crystal structure has been deposited with the Protein Data bank under accession number 7NX5. NMR data have been deposited in the BMRB database under accession numbers 50847 and 50848.

## Supplementary Material

gkab1183_Supplemental_File
