# Supplementary material for: Structural basis of DNA methylation-dependent site selectivity of the Epstein–Barr virus lytic switch protein ZEBRA/Zta/BZLF1
Source: Nucleic Acids Res. 2021 Dec 10;50(1):490–511. doi: 10.1093/nar/gkab1183 (PMC8754650; doi:10.1093/nar/gkab1183)
Supplement: gkab1183_Supplemental_File [file gkab1183_supplemental_file.pdf]

Structural basis of DNA methylation-dependent site selectivity of the Epstein-Barr virus lytic switch protein ZEBRA/Zta/BZLF1

Bernaudat et al.

Supplementary Data

Supplementary Table 1. Crystallographic data collection and refinement statistics.

**Data Collection**		
ESRF beamline		ID23-2
Wavelength (Å)		0.8726
Space group		C2
Unit cell dimensions		*a*=208.0 Å, *b*=26.56 Å, *c*=80.85 Å, β=103.1°
No. complexes in asymmetric unit		2
Resolution range (Å) [1]		34 – 2.5 (2.6 – 2.5)
No. of measured reflections		56,118 (6368)
No. of unique reflections		15,447 (1710)
Multiplicity		3.63 (3.72)
Completeness (%)		98.9 (99.8)
Mean I/sigma(I)		12.7 (2.4)
R_{meas}		0.082 (0.712)
$CC_{1/2}$		0.998 (0.767)
**Refinement**		
Resolution		16 – 2.5
No. reflections (total/R_{free})		14,841 / 762
R_{work}/R_{free}		0.2768 / 0.3387
Number of atoms/Mean *B*-factor (Å^2)		
Protein:	Complex 1 (chains A,B)	992 / 52.0
	Complex 2 (chains E,F)	953 / 69.6
	Total	1996 / 60.8
DNA:	Complex 1 (chains C,D)	650 / 66.0
	Complex 2 (chains G,H)	501 / 96.4
	Total	1164 / 79.3
Water:		65 / 40.6
R.m.s. deviations:		
Bond distances (Å)		0.010
Bond angles (°)		1.159
Ramachandran analysis (%)		
Favored/ outliers		96.6 / 0.0
Molprobity analysis		
Clash Score / Overall score		12.92 / 2.27

[1] Numbers in parentheses refer to the outer resolution shell.

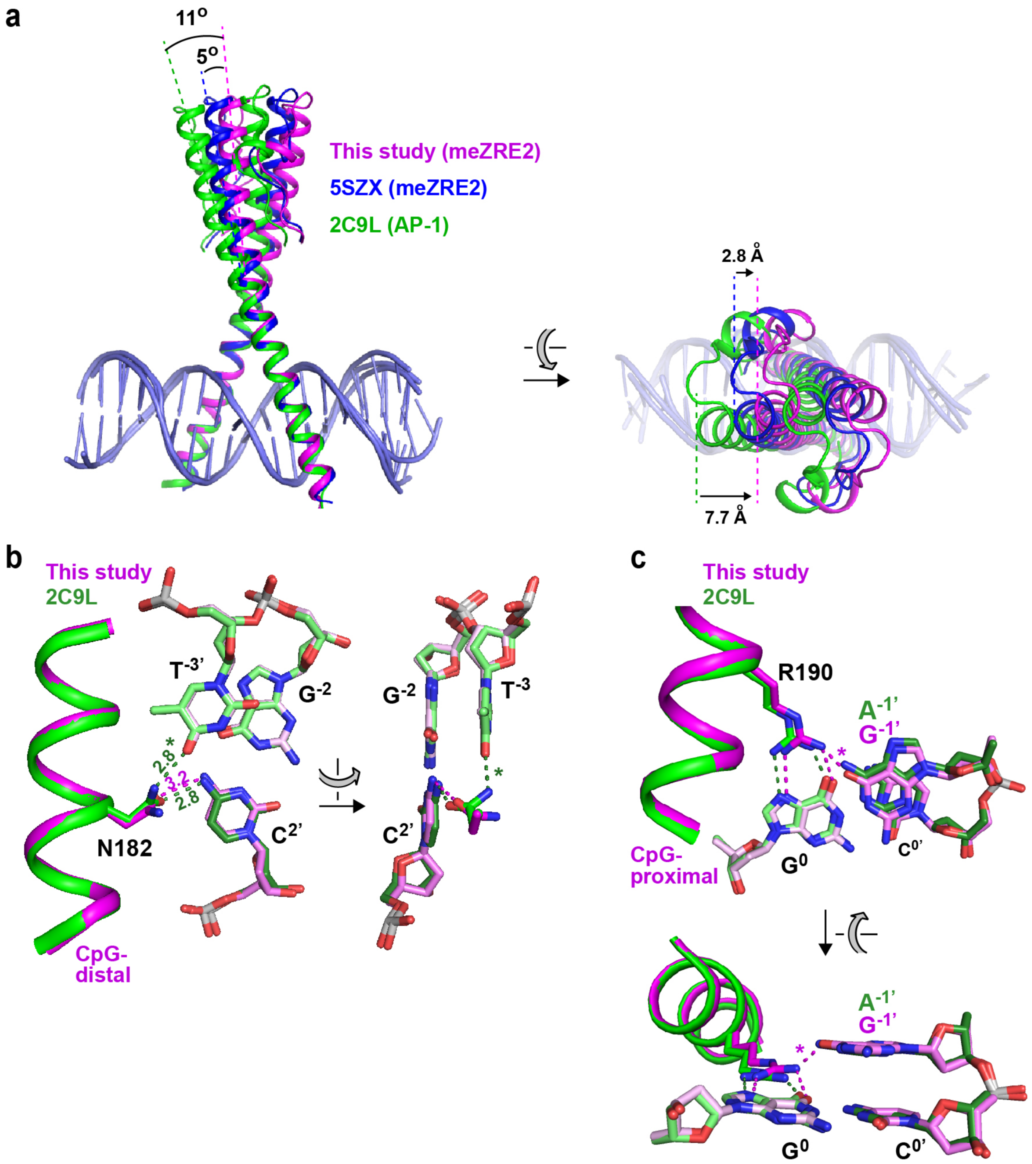

Figure S1. Comparison of ZEBRA/DNA complex structures.

a. Previous structures of ZEBRA bound to an AP-1 site (PDB 2C9L) and to the Rp meZRE2 site (PDB 5SZX) were aligned with our crystal structure via the basic region (res. 180-200) and the heptad DNA site (base pairs -3 to +3) , yielding overall RMSD values (for 122 Cα and 30 P atoms) of 1.73 and 0.91 Å, respectively. These values reduce to 0.43 and 0.33 Å when only the bZIP basic regions and 7-bp binding site (42 Cα and 14 P atoms) are compared, highlighting that the structures mainly differ in the dimerization domain. The latter domain exhibits a variable degree of bending by up to 11°, corresponding to a relative displacement of ~8 Å at the tip of the coiled coil.

b. Comparison of interactions between Asn^{182} and the A half-sites of AP-1 and meZRE2. ZEBRA interacts with the meZRE2 A half-site essentially as in the ZEBRA/AP-1 structure (PDB 2C9L) except for contacts mediated by residue Asn^{182}. In the AP-1 complex Asn^{182} forms bridging H-bonds with the $C^{2'}$ and T^{-3} bases, whereas in the meZRE2 complex a slightly rotated Asn^{182} side chain interacts only with meZRE2 base $C^{2'}$ and forms a weaker H-bond than in the AP-1 complex (bond distance of 3.2 Å versus 2.8 Å). However, in the alternate crystal form of Hong et al. (42) Asn^{182} adopts the same conformation as in the AP-1 complex, suggesting that ZEBRA tolerates variability of the Asn^{182} rotamer when bound to meZRE2. DNA bases of AP-1 are shown in light and dark green, those of meZRE2 in pink and violet. H-bond lengths are indicated in Ångstroms. The green asterisk indicates the H-bond interaction absent in our crystal structure.

c. CpG-proximal Arg^{190}-mediated interactions in the AP-1 and meZRE2 complexes. Coloring scheme is as in (b). The magenta asterisk indicates the additional interaction (part of a bifurcated H-bond) observed in the meZRE2 complex that is absent from the AP-1 complex since the $A^{-1'}$ exocyclic N6 atom cannot accept a proton.

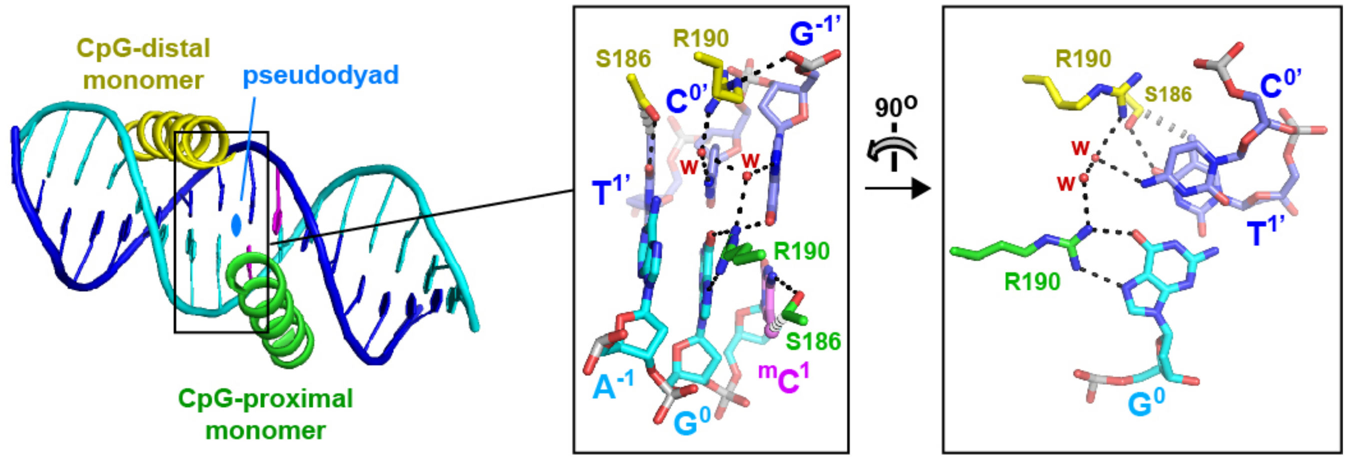

Figure S2. DNA interactions and hydrogen bond network involving ZEBRA's CpG-distal and CpG-proximal Arg^{190} residues. H-bonds are shown as dashed black lines and van der Waals contacts as thick broken gray lines. Two water molecules (labeled "w") bridge the A and M half sites by forming H-bonds with each other, with the C$^{0'}$ and G$^{-1'}$ bases, and with the guanidino groups of the two Arg^{190} residues.

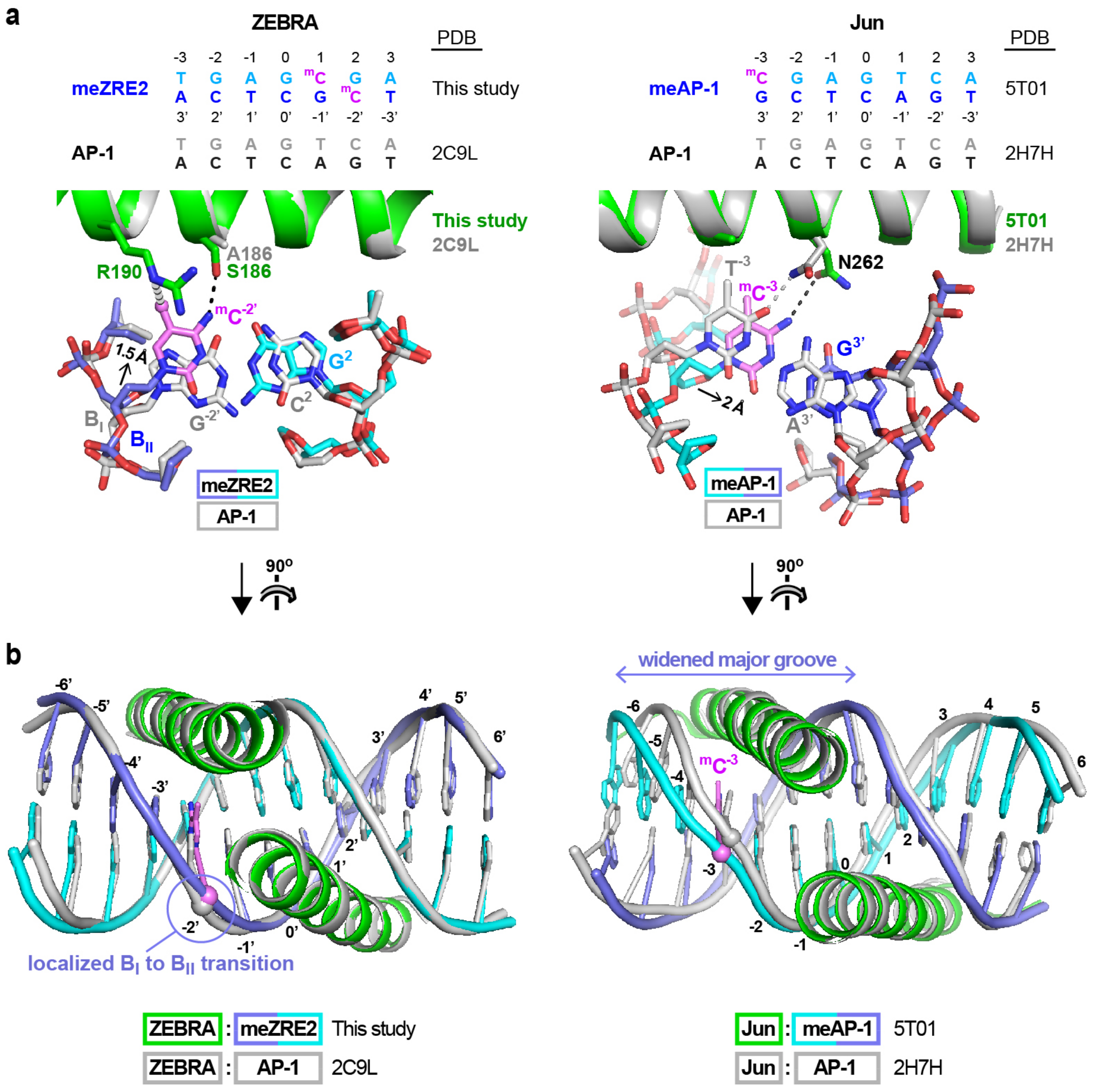

Figure S3. Comparison of meZRE2, AP-1 and meAP-1 DNA conformations bound to ZEBRA and Jun.

a. Displacements involving methylcytosines -2' of ZEBRA-bound meZRE2 (left) and -3 of Jun-bound meAP-1 (right) relative to the corresponding AP-1 nucleotide. Black and gray dashed lines indicate hydrogen bond and van der Waals interactions, respectively.

Left. meZRE2 nucleotide $^{m}C^{-2'}$ is shifted by 1.5 Å relative to AP-1. This displacement cannot be ascribed to the use of a ZEBRA mutant to solve the AP-1 bound structure (43) since a lower resolution structure of wildtype (WT) ZEBRA bound to AP-1 (PDB 2C9N) exhibits the identical position for the $G^{-2'}$ base, and because the corresponding G^{-2} base in the A half-site of meZRE2 adopts the same unshifted position. *Right.* Aligning the structures of Jun bound to AP-1 and to a variant (meAP-1) containing a methylcytosine at position -3 shows that the $^{m}C^{-3}$ base of meAP-1 is displaced by 2 Å relative to the T^{-3} base of AP-1 (42). The shift occurs in a direction orthogonal to that observed in the ZEBRA/meZRE2 complex and allows $^{m}C^{-3}$ to maintain a hydrogen bond with the altered side chain conformation of Jun residue Asn^{262}. (The atomic coordinates of 2H7H were modified by flipping the chi2 angle of Asn^{262} by 180° so that the H-bonding geometry makes sense). Note that the left and right panels, shown in the same relative orientation to facilitate comparison, concern nucleotide shifts in the right and left DNA half-sites, respectively.

b. Structural alignment of the ZEBRA-bound meZRE2 and AP-1 complexes (left) compared with that of the Jun-bound AP-1 and meAP-1 complexes (right). Whereas the conformational change in the DNA backbone is restricted to the -2' nucleotide on the left, it extends over several nucleotides on the right.

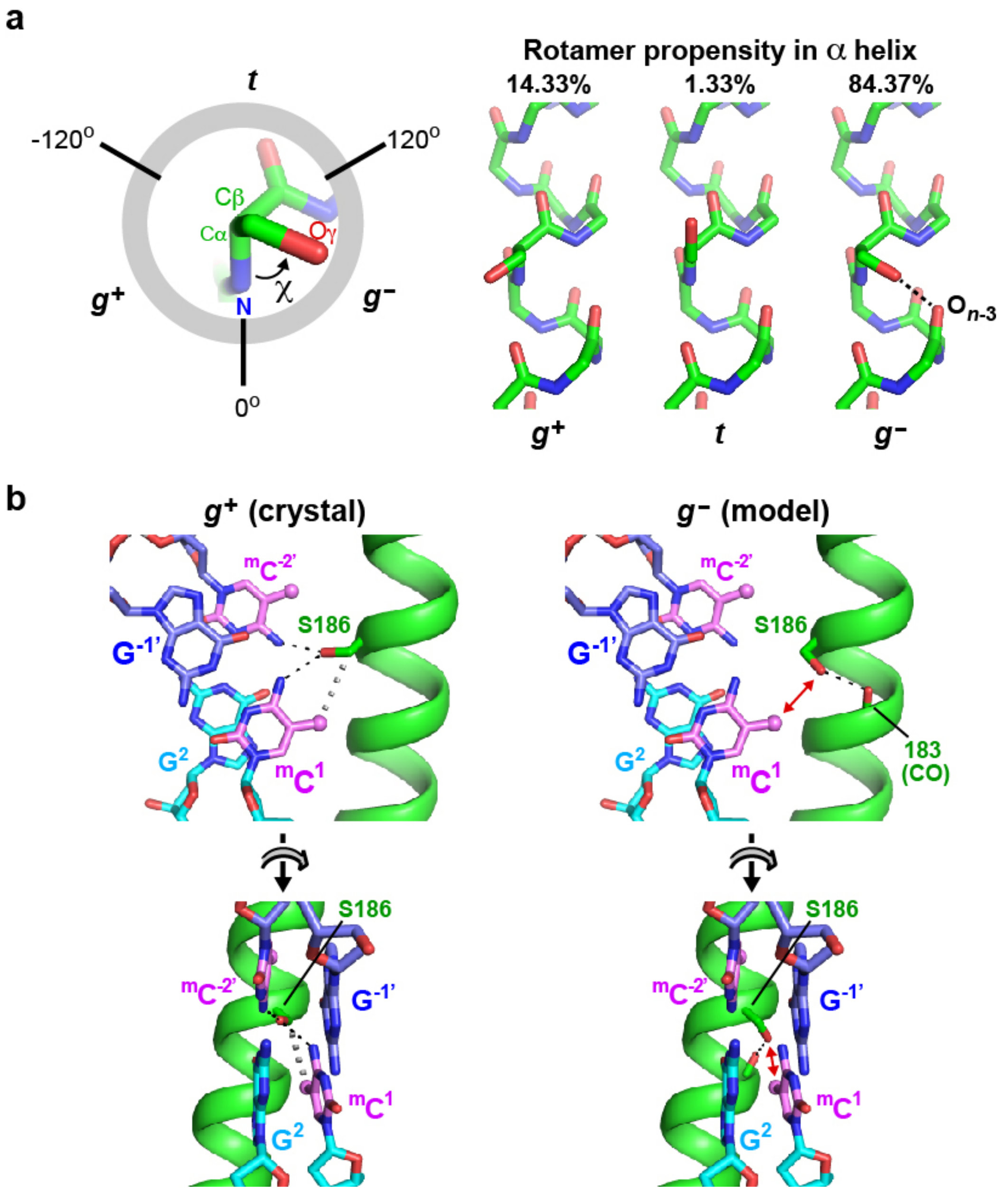

Figure S4. Stabilization of the Ser[186] g^{+} rotamer by the $^{m}C^{1}$ methyl group.

a. *Left,* Torsion angle convention for serine showing the g^{+}, g^{-} and t rotamers. *Right,* Propensities for serine to adopt the three different rotamers when located within an α helix. The g^{-} conformation is highly favoured because it allows the Ser hydroxyl group to hydrogen bond with the carbonyl group of residue *N*-3. Propensities are from ref. (77).

b. Comparison between the g^{+} and g^{-} rotamers in the presence of meZRE2. Modeling the g^{-} rotamer of Ser[186] in the ZEBRA/meZRE2 complex shows that this rotamer unfavorably juxtaposes the polar Ser[186] hydroxyl and hydrophobic $^{m}C^{1}$ methyl groups (red arrows). Black and gray dashed lines indicate H-bonds and van der Waals contacts, respectively.

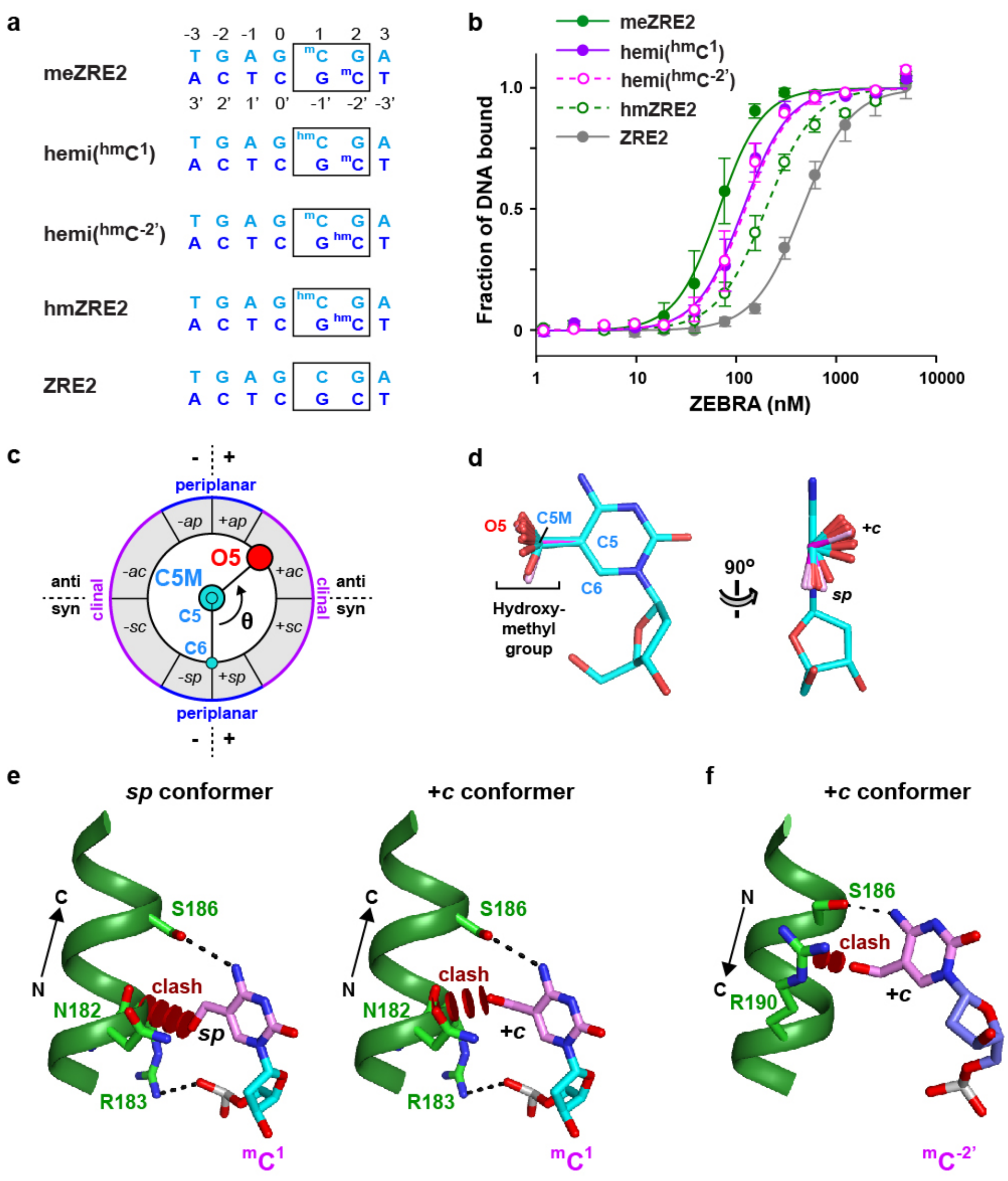

Figure S5. CpG hydroxymethylation destabilizes the ZEBRA/meZRE2 complex.

a. ZRE2 sequences used for binding assays. Cytosine nucleotides 1 and -2' were either unmodified, methylated or hydroxymethylated as indicated.

b. FP assays assessing the binding of ZEBRA to ZRE2 sites that are either unmodified, symmetrically methylated or hydroxymethylated, or that bear one hydroxymethyl and one methyl mark. The data shown represent the mean ± SD from three independent experiments.

c. Torsion angle definition for the hydroxymethyl group of ^{hm}C. The view is along the C5M-C5 bond, with the C5M atom closer to the viewer.

d. Alignment of ^{hm}C nucleotides from PDB entries containing B-form DNA. A total of 18 nucleotides from 11 high-resolution (better than 2 Å) crystal structures were aligned (PDB entries 4glc, 4glh, 4hli, 4i9v, 4pba, 4pbB, 4r2c, 4r2p, 5cjy, 5deu, 5dsb). For clarity, non-hydroxymethyl group atoms are shown for only one nucleotide. The alignment reveals two predominant orientations for the hydroxymethyl group, syn-periplanar (*sp*; θ=-14° to 25°) and +clinal (+*c*; θ=73° to 135°). Hydroxymethyl groups from structures in which the CpG motif is symmetrically hydroxymethylated on both cytosines (PDB entries 4r2c and 4r2p) are shown with their C5 and C5M atoms in magenta and their O5 atom in pink.

e,f. Model of the ZEBRA/meZRE2 complex with (e) ^{m}C^{1} and (f) ^{m}C$^{-2'}$ replaced by ^{hm}C**.** Representative *sp* (θ=15°) and +*c* (θ=109°) conformations are taken from a symmetrically hydroxymethylated CpG motif (pdb 4r2p, ^{hm}C residues B9 and C53, respectively). Hydrogen bonds and steric clashes are indicated by dashed black lines and by a series of red disks, respectively.

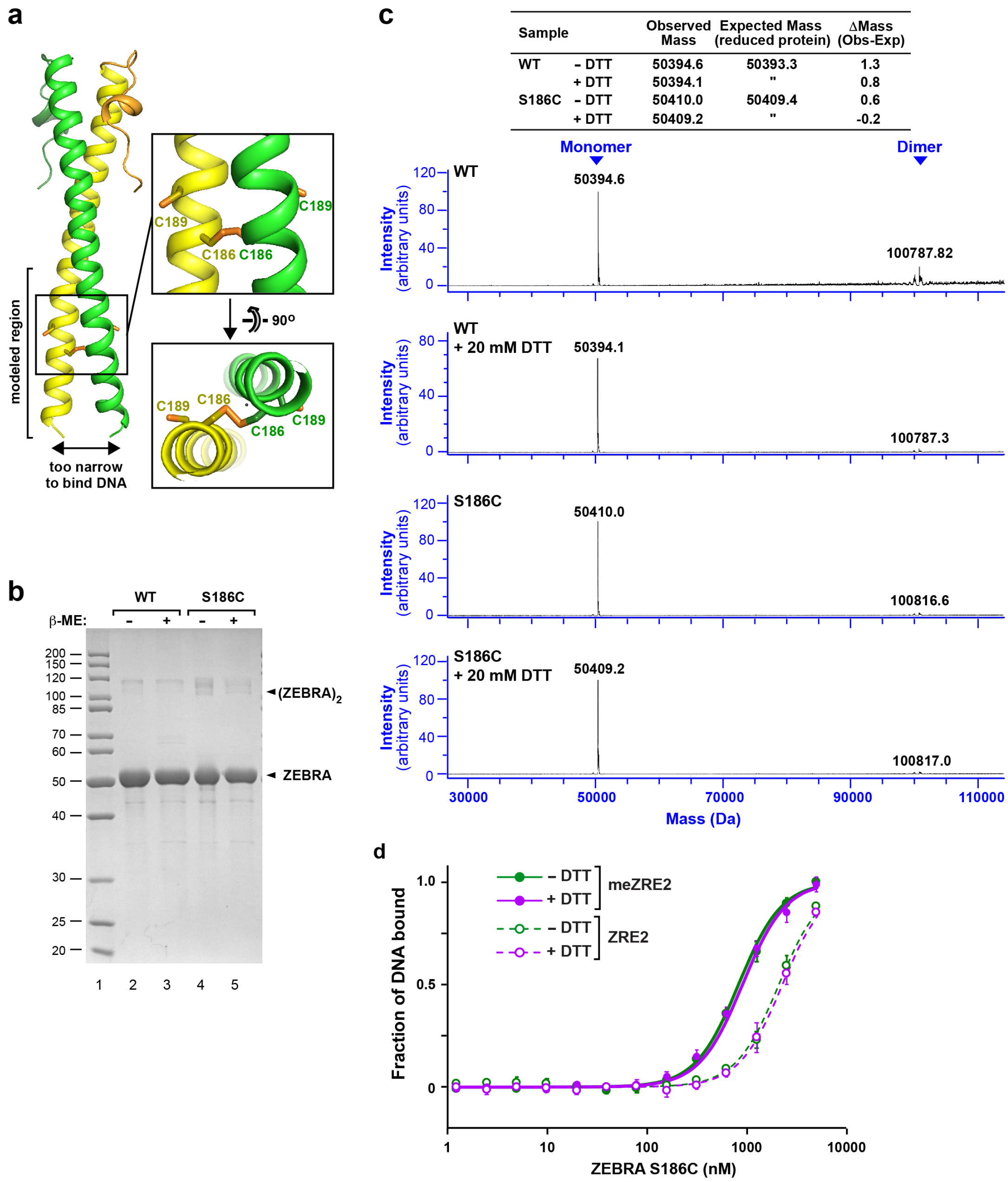

Figure S6. Poor DNA-binding activity of the ZEBRA S186C mutant is not due to cysteine oxidation. a. Hypothetical model of a disulfide-crosslinked homodimer formed by ZEBRA mutant S186C. Because residue 186 localizes to the inner helical surface of each monomer in the ZEBRA homodimer, the two Cys^{186} residues could hypothetically form a disulfide crosslink that would lock the homodimer in a conformation incompatible with DNA binding. (In contrast, the two Cys^{189} residues are too far apart to spontaneously form a disulfide bond). The indicated modeled region was obtained by extending the helical trajectory of ZEBRA's zipper region towards the N-terminus using a canonical coiled coil (PDB 3HE5) as a template (118). **b.** Coomassie-stained SDS denaturing gel showing that the amount of covalent ZEBRA homodimer formation is negligible. WT and mutant forms of MBP-tagged ZEBRA proteins used for FP assays were heated at 95°C for 5 min in the presence (lanes 3 and 5) or absence (lanes 2 and 4) of β-mercaptoethanol (β-ME; 180 mM) prior to loading on the gel. **c.** Mass spectrometry (LC/ESI) analysis of WT and mutant MBP-tagged ZEBRA proteins used for FP assays. Proteins were incubated in the presence or absence of 20 mM dithiothreitol (DTT) prior to analysis. The measured masses of the monomers closely match the theoretical expected mass, confirming that cysteine residues are in their fully reduced state. Only trace amounts of covalent ZEBRA homodimer are detected, indicating that there is little disulfide crosslink formation. **d.** FP assays assessing the binding of the S186C mutant to the meZRE2 site. Protein samples were incubated in the presence or absence of 10 mM DTT prior to performing the assay. The presence of the reducing agent had no detectable effect on the DNA-binding activity of the S186C mutant.

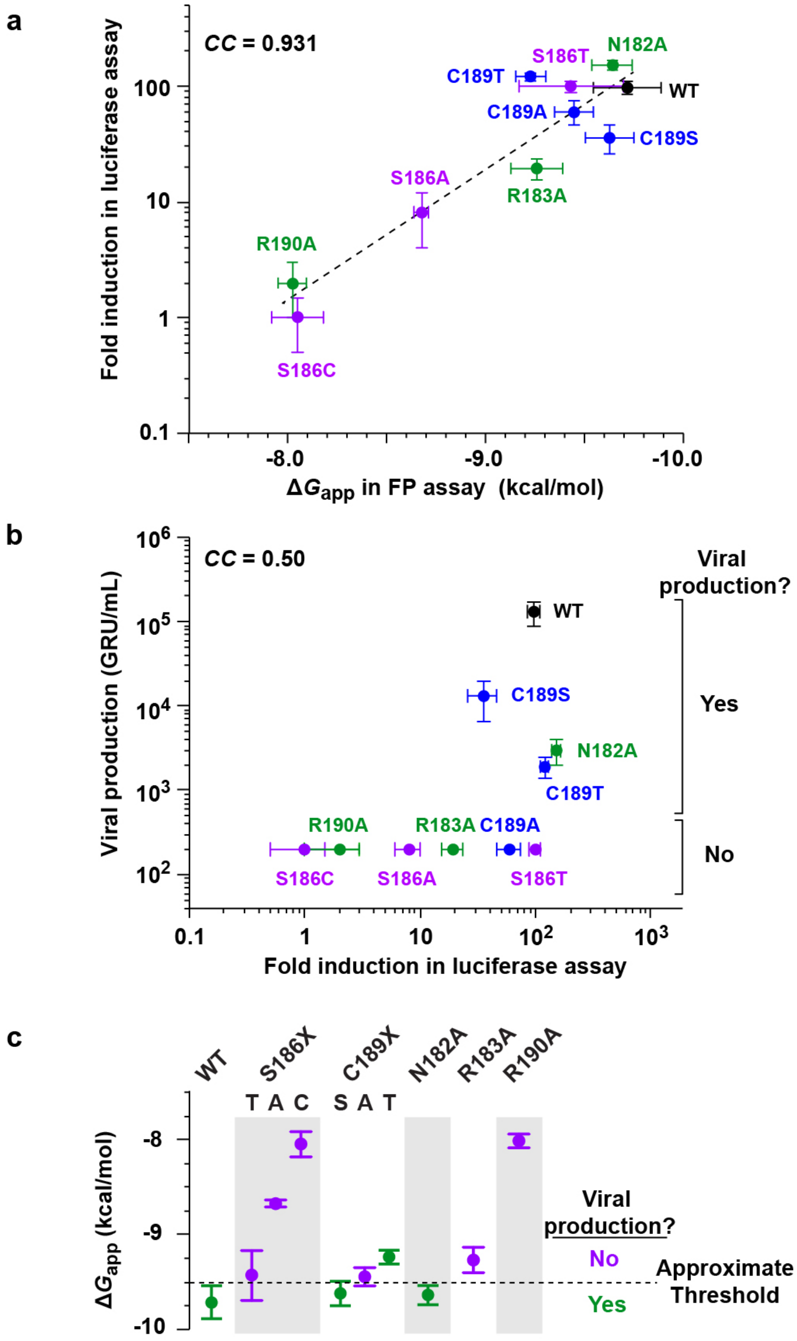

Figure S7. Transactivation of a CpG-methylated promoter mirrors stability of the ZEBRA/meZRE2 complex whereas viral production shows a threshold-like response.

a. Fold-induction in luciferase reporter assays plotted against the apparent free binding energy measured in FP assays. The correlation coefficient (*CC*) between the log of the fold induction and ΔG_{app} is indicated.

b. Viral production plotted against fold-induction in the luciferase reporter assay. The *CC* between the log of viral production and the log of fold induction is indicated. GRU, Green Raji Units.

c. The ability to induce viral production is associated with ZEBRA/meZRE2 complex stability. Point mutants that induced or failed to induce detectable viral production are shown in green and magenta, respectively.

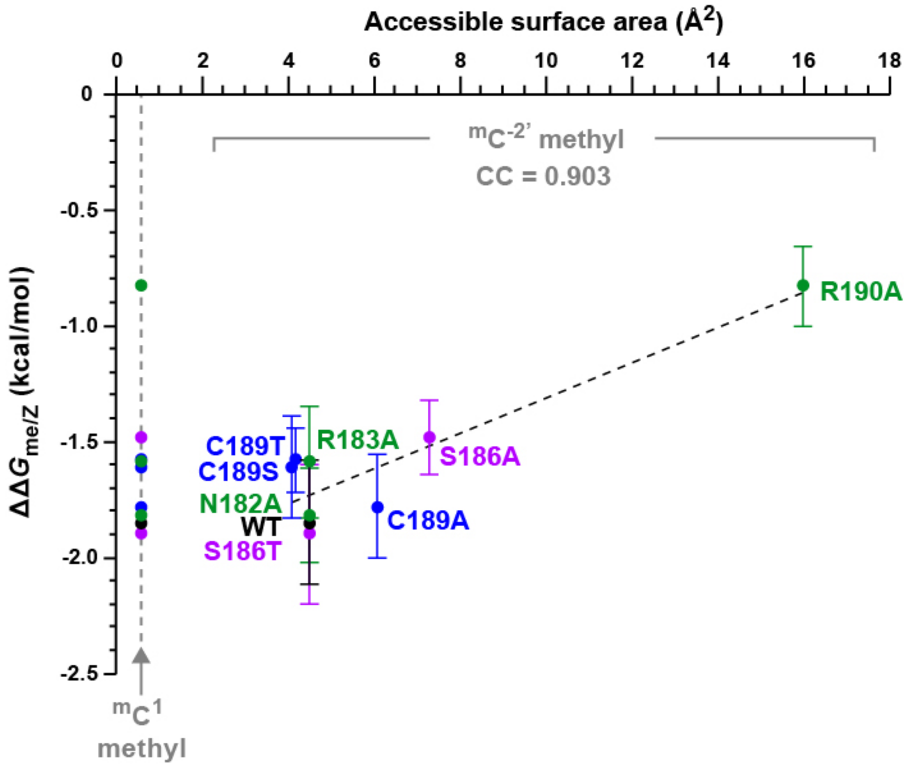

Figure S8. Stabilization induced by CpG methylation (ΔΔ$G_{me/Z}$) plotted against solvent accessibility of the CpG methylation marks.

Structures of meZRE2-bound ZEBRA mutants were modeled and the accessible surface area (ASA) of the cytosine methyl groups was calculated using the Areaimol program from the CCP4 suite (46). The crystal structure of the ZEBRA/meZRE2 complex was modified by replacing the mutated residue by alanine or, in the case of non-alanine subtitutions, by selecting the sterically allowed rotamer most closely resembling that of the WT residue. In the case of the S186A mutant the ^{m}C$^{-2'}$ nucleotide was modeled in the B_I conformation based on the crystal structure of the S186A mutant bound to AP-1 (PDB 2C9L), since loss of the serine hydroxyl group would destabilize the B_{II} conformation (**Figure 2**). None of the point mutations altered the solvent exposure of the methyl group on ^{m}C^1, which remained highly buried (ASA=0.6 Å^2). Not shown is the S186C mutant which, with the same ASA for the ^{m}C$^{-2'}$ group as WT ZEBRA and a ΔΔ$G_{me/Z}$ of -1.08 kcal/mol, appeared as a clear outlier in this plot.

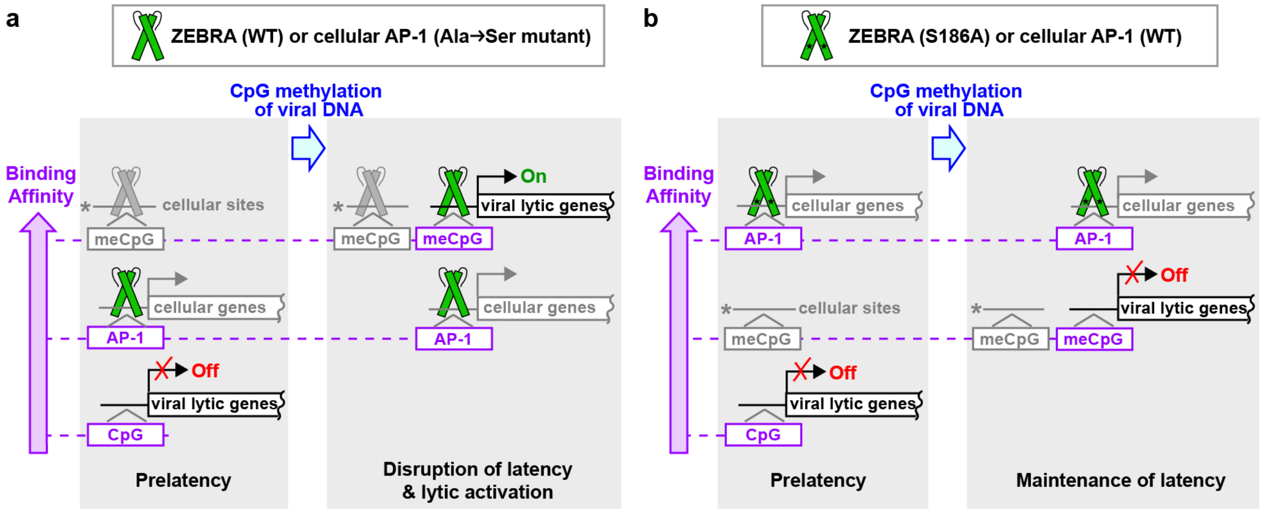

Figure S9. ZEBRA's transactivation activity depends on two types of target site selectivity.

a. Schematic illustration showing the dual transactivation roles of ZEBRA during the EBV infection cycle. ZEBRA's binding sites are vertically arranged in order of increasing affinity. Methylated CpG-containing ZREs in the host cell genome are marked by an asterisk and shown in gray. ZEBRA homodimers bound to these sites are also in gray. AP-1 sites within the viral genome are omitted for clarity. During prelatency when the EBV genome is unmethylated the ZEBRA homodimer preferentially binds to AP-1 sites over non-methylated CpG-containing sites. During latency, ZEBRA binds to methylated CpG-containing ZREs within viral lytic promoters, leading to the disruption of viral latency. Similar site selectivity by a cellular AP-1 dimer bearing a gain-of-function Ala→Ser mutation also leads to viral lytic gene expression (41).

b. Inability of ZEBRA mutant S186A to disrupt viral latency. The S186A mutation renders ZEBRA's site selectivity similar to that of cellular AP-1 proteins, which preferentially bind AP-1 sites over methylated and unmethylated CpG-containing ZREs. Sequestration by the higher-affinity AP-1 sites prevents cellular AP-1 and mutant ZEBRA proteins from binding the methylated ZREs and activating viral lytic expression.

a

EBV strain	Origin		K	N	R	V	A	S (186)	R	K	C	R	A		GenBank ID
B95-8/Raji	USA & Nigeria	102491	AAG	AAT	CGG	GTG	GCT	TCC	AGA	AAA	TGC	CGG	GCC	102459	V01555
GD1	China	90093	AAG	AAT	CGG	GTG	GCT	TCC	AGA	AAA	TGC	CGG	GCC	90061	AY961628
GD2	China	87765	AAG	AAT	CGG	GTG	GCT	TCC	AGA	AAA	TGC	CGG	GCC	87733	HQ020558
AG876	Ghana	91006	AAG	AAT	CGG	GTG	GCT	TCC	AGA	AAA	TGC	CGG	GCC	90974	DQ279927
HKNPC1	Hong Kong	89827	AAG	AAT	CGG	GTG	GCT	TCC	AGA	AAA	TGC	CGG	GCC	89795	JQ009376
Akata	Japan	89863	AAG	AAT	CGG	GTG	GCT	TCC	AGA	AAA	TGC	CGG	GCC	89831	KC207813
Mutu	Kenya	90073	AAG	AAT	CGG	GTG	GCT	TCC	AGA	AAA	TGC	CGG	GCC	90041	KC207814
M81	Hong Kong	90245	AAG	AAT	CGG	GTG	GCT	TCC	AGA	AAA	TGC	CGG	GCC	90213	KF373730
K4123-mi	USA	90211	AAG	AAT	CGG	GTG	GCT	TCC	AGA	AAA	TGC	CGG	GCC	90179	KC440851
K4413-Mi	USA	90221	AAG	AAT	CGG	GTG	GCT	TCC	AGA	AAA	TGC	CGG	GCC	90189	KC440852

b

bZIP Protein	E-value														GenBank ID
CREM	2e-09	195	K<br>AAA	N<br>AAC	R<br>AGG	E<br>GAA	A<br>GCT	A<br>GCC	K<br>AAA	E<br>GAA	C<br>TGT	R<br>CGA	R<br>CGT	205	XM_011519332
C/EBP-delta	3e-08	200	R<br>CGC	N<br>AAC	N<br>AAC	I<br>ATC	A<br>GCC	V<br>GTG	R<br>CGC	K<br>AAG	S<br>AGC	R<br>CGC	D<br>GAC	210	M83667
ATF-1	3e-08	128	K<br>AAA	N<br>AAC	R<br>AGA	E<br>GAA	A<br>GCT	A<br>GCT	R<br>CGA	E<br>GAA	C<br>TGT	R<br>CGC	R<br>AGA	138	XM_011538388
XBP-1	4e-08	79	K<br>AAA	N<br>AAC	R<br>AGA	V<br>GTA	A<br>GCA	A<br>GCT	Q<br>CAG	T<br>ACT	A<br>GCC	R<br>AGA	D<br>GAT	89	NM_001079539
CREB1	4e-08	238	K<br>AAG	N<br>AAC	R<br>AGG	E<br>GAA	A<br>GCA	A<br>GCT	R<br>CGA	E<br>GAG	C<br>TGT	R<br>CGT	R<br>AGA	248	NM_001371428
CREBRF	1e-07	530	K<br>AAA	N<br>AAT	K<br>AAG	L<br>CTG	A<br>GCT	S<br>TCC	R<br>AGA	A<br>GCT	C<br>TGT	R<br>CGG	L<br>TTA	540	NM_153607
ATF2	2e-07	255	R<br>CGA	N<br>AAT	R<br>AGA	A<br>GCA	A<br>GCA	A<br>GCT	S<br>TGA	R<br>AGA	C<br>TGC	R<br>CGA	Q<br>CAA	265	DQ003042
JunB	2e-07	277	R<br>CGG	N<br>AAC	R<br>CGG	L<br>CTG	A<br>GCG	A<br>GCC	T<br>ACC	K<br>AAG	C<br>TGC	R<br>CGG	K<br>AAG	287	NM_002229
CRE-BP1	4e-07	185	R<br>CGA	N<br>AAT	R<br>AGA	A<br>GCA	A<br>GCA	A<br>GCT	S<br>TCA	R<br>AGA	C<br>TGC	R<br>CGA	Q<br>CAA	195	U16028.1
ATF-7	4e-07	286	R<br>CGC	N<br>AAC	R<br>CGG	A<br>GCT	A<br>GCA	A<br>GCC	S<br>TCC	R<br>CGC	C<br>TGC	R<br>CGC	Q<br>CAA	296	NM_001366561
JunD	2e-06	277	R<br>CGC	N<br>AAC	R<br>CGC	I<br>ATC	A<br>GCC	A<br>GCC	S<br>TCC	K<br>AAG	C<br>TGC	R<br>CGC	K<br>AAG	287	X56681
C/EBPbeta	4e-06	280	R<br>CGC	N<br>AAC	N<br>AAC	I<br>ATC	A<br>GCC	V<br>GTG	R<br>CGC	K<br>AAG	S<br>AGC	R<br>CGC	D<br>GAC	290	NM_005194

Figure S10. Comparison of ZEBRA and human bZIP sequences in the vicinity of residue 186.

a. Alignment of genomic DNA sequences from representative EBV strains. ZEBRA residue Ser^{186} is invariant and specified by a TCC codon in all known EBV strains, as revealed by a BLAST alignment of all EBV sequences in the NCBI nucleotide collection (1084 entries). Origin of strains is from ref. (119).

b. Alignment of nucleotide and protein sequences from human bZIP proteins closely related to ZEBRA. Human proteins are ordered according to descending E-score from a BLAST search performed with the basic and coiled-coil regions of ZEBRA (residues 175-221) against all human proteins in the non-redundant protein database using the DELTA-BLAST algorithm.

bZIP Protein		186		UniProt ID
ZEBRA	175	LEIKRYKNRVASRKCRAKFKQLLQHYREVAAAKSSENDRLRLLLKQM	221	P03206
CREBRF	524	PRSRKEKNKLASRACRLKKKAQYEANKVKLWGLNTEYDNLLFVINSI	570	Q8IUR6
CEBPA	285	YRVRRERNNIAVRKSRDKAKQRNVETQQKVLELTSDNDRLRKRVEQL	331	P49715
CEBPB	274	YKIRRERNNIAVRKSRDKAKMRNLETQHKVLELTAENERLQKKVEQL	320	P17676
CEBPG	65	YRQRRERNNMAVKKSRLKSKQKAQDTLQRVNQLKEENERLEAKIKLL	111	P53567
CEBPD	194	YRQRRERNNIAVRKSRDKAKRRNQEMQQKLVELSAENEKLHQRVEQL	240	P49716
CEBPE	207	YRLRRERNNIAVRKSRDKAKRRILETQQKVLEYMAENERLRSRVEQL	253	Q15744
CREM	289	RELRLMKNREAAKECRRRKKEYVKCLESRVAVLEVQNKKLIEELETL	335	Q03060
CREB1	286	REVRLMKNREAARECRRKKKEYVKCLENRVAVLENQNKTLIEELKAL	332	P16220
CREBL2	26	LKAKLERSRQSARECRARKKLRYQYLEELVSSRERAICALREELEMY	72	O60519
CREB3	153	RVRRKIRNKRSAQESRRKKKVYVGGLESRVLKYTAQNMELQNKVQLL	199	O43889
CREB3L1	293	RVRRKIKNKISAQESRRKKKEYVECLEKKVETFTSENNELWKKVETL	340	Q96BA8
CREB3L2	297	KIRRKIKNKISAQESRRKKKEYMDSLEKKVESCSTENLELRKKVEVL	343	Q70SY1
CREB3L3	246	KIRRKIRNKQSAQESRKKKKEYIDGLETRMSACTAQNQELQRKVLHL	292	Q68CJ9
CREB3L4	220	KVRRKIRNKQSAQDSRRRKKEYIDGLESRVAACSAQNQELQKKVQEL	266	Q8TEY5
CREB5	378	RRKFLERNRAAATRCRQKRKVWVMSLEKKAEELTQTNMQLQNEVSML	424	Q02930
NFIL3	76	YWEKRRKNNEAAKRSREKRRLNDLVLENKLIALGEENATLKAELLSL	122	Q16649
NFILZ	45	YWEKRRKNNEAAKRSREKRRLNDAAIEGRLAALMEENALLKGELKAL	91	A0A5F9ZHS7
TEF	236	YWTRRKKNNVAAKRSRDARRLKENQITIRAAFLEKENTALRTEVAEL	282	Q10587
DBP	258	YWSRRYKNNEAAKRSRDARRLKENQISVRAAFLEKENALLRQEVVAV	304	Q10586
HLF	228	YWARRRKNNMAAKRSRDARRLKENQIAIRASFLEKENSALRQEVADL	274	Q16534
CREBZF	207	TKSPRKAAAAAARLNRLKKKEYVMGLESRVRGLAAENQELRAENREL	253	Q9NS37
DDIT3	102	TRKRKQSGHSPARAGKQRMKEKEQENERKVAQLAEENERLKQEIERL	148	P35638
JDP2	75	RKRRREKNKVAAARCRNKKKERTEFLQRESERLELMNAELKTQIEEL	121	Q8WYK2
ATF1	216	REIRLMKNREAARECRRKKKEYVKCLENRVAVLENQNKTLIEELKTL	262	P18846
ATF2	355	RRKFLERNRAAASRCRQKRKVWVQSLEKKAEDLSSLNGQLQSEVTLL	401	P15336
ATF3	89	KKRRRERNKIAAAKCRNKKKEKTECLQKESEKLESVNAELKAQIEEL	135	P18847
ATF4	281	KLKKMEQNKTAATRYRQKKRAEQEALTGECKELEKKNEALKERADSL	327	P18848
ATF5	211	KQKKRDQNKSAALRYRQRKRAEGEALEGECQGLEARNRELKERAESV	257	Q9Y2D1
ATF6A	309	RQQRMIKNRESACQSRKKKKEYMLGLEARLKAALSENEQLKKENGTL	355	P18850
ATF6B	328	RQQRMIKNRESACQSRRKKKEYLQGLEARLQAVLADNQQLRRENAAL	374	Q99941
ATF7	346	RQRFLERNRAAASRCRQKRKLWVSSLEKKAEELTSQNIQLSNEVTLL	392	P17544
BATF	29	RVQRREKNRIAAQKSRQRQTQKADTLHLESEDLEKQNAALRKEIKQL	75	Q16520
BATF2	20	RQLKKQKNRAAAQRSRQKHTDKADALHQQHESLEKDNLALRKEIQSL	66	Q8N1L9
BATF3	38	KVRRREKNRVAAQRSRKKQTQKADKLHEEYESLEQENTMLRREIGKL	84	Q9NR55
FOS	140	RRIRRERNKMAAAKCRNRRRELTDTLQAETDQLEDEKSALQTEIANL	186	P01100
FOSB	158	RRVRRERNKLAAAKCRNRRRELTDRLQAETDQLEEEKAELESEIAEL	204	P53539
FOSL1	108	RRVRRERNKLAAAKCRNRRKELTDFLQAETDKLEDEKSGLQREIEEL	154	P15407
FOSL2	127	RRIRRERNKLAAAKCRNRRRELTEKLQAETEELEEEKSGLQKEIAEL	173	P15408
JUN	255	AERKRMRNRIAASKCRKRKLERIARLEEKVKTLKAQNSELASTANML	301	P05412
JUNB	271	VERKRLRNRLAATKCRKRKLERIARLEDKVKTLKAENAGLSSTAGLL	317	P17275
JUND	271	AERKRLRNRIAASKCRKRKLERISRLEEKVKTLKSQNTELASTASLL	317	P17535
XBP1	73	ALRRKLKNRVAAQTARDRKKARMSELEQQVVDLEEENQKLLLENQLL	119	P17861
BACH1	560	DIRRRSKNRIAAQRCRKRKLDCIQNLESEIEKLQSEKESLLKERDHI	606	O14867
BACH2	649	DVRRRSKNRIAAQRCRKRKLDCIQNLECEIRKLVCEKEKLLSERNQL	695	Q9BYV9
NFE2	269	DIRRRGKNKVAAQNCRKRKLETIVQLERELERLTNERERLLRARGEA	315	Q16621
NFE2L1	657	DIRRRGKNKMAAQNCRKRKLDTILNLERDVEDLQRDKARLLREKVEF	703	Q14494
NFE2L2	500	DIRRRGKNKVAAQNCRKRKLENIVELEQDLDHLKDEKEKLLKEKGEN	546	Q16236
NFE2L3	581	DIRRRGKNKVAAQNCRKRKLDIILNLEDDVCNLQAKKETLKREQAQC	627	Q9Y4A8
MAF	291	QKRRTLKNRGYAQSCRFKRVQQRHVLESEKNQLLQQVDHLKQEISRL	337	O75444
MAFA	257	QKRRTLKNRGYAQSCRFKRVQQRHILESEKCQLQSQVEQLKLEVGRL	303	Q8NHW3
MAFB	241	QKRRTLKNRGYAQSCRYKRVQQKHHLENEKTQLIQQVEQLKQEVSRL	287	Q9Y5Q3
MAFG	54	QRRRTLKNRGYAASCRVKRVTQKEELEKQKAELQQEVEKLASENASM	100	O15525
MAFF	54	QRRRTLKNRGYAASCRVKRVCQKEELQKQKSELEREVDKLARENAAM	100	Q9ULX9
MAFK	54	QRRRTLKNRGYAASCRIKRVTQKEELERQRVELQQEVEKLARENSSM	100	O60675
NRL	162	QRRRTLKNRGYAQACRSKRLQQRRGLEAERARLAAQLDALRAEVARL	208	P54845

Figure S11. ZEBRA residue Ser^{186} corresponds to a nearly invariant alanine in human bZIP proteins. Sequence alignment of ZEBRA's basic and coiled coil residues with the corresponding regions of the 55 unique human bZIP proteins available in the UniProt database. ZEBRA residue Ser^{186} corresponds to an alanine in all these proteins except for C/EBP protein family members, which have a valine, and CREBRF, which has a serine like ZEBRA. CREBRF negatively regulates CREB3, a bZIP protein implicated in the endoplasmic reticulum stress response, by sequestering it in discrete nuclear foci and promoting its degradation (120). CREBRF also represses glucocorticoid stress signaling (121), inhibits lytic replication of herpes simplex virus 1 (122) and is implicated in diverse developmental and cancer-related processes (123-125). The CREBRF *Drosophila* ortholog REPTOR (which also has the serine) associates with the bZIP protein REPTOR-BP to mediate the transcriptional response to the cellular energy sensor Target of Rapamycin Complex 1 (TORC1)(115) ChIP-Seq analysis identified potential REPTOR/REPTOR-RB binding sites that contain CpG motifs, including one within a known REPTOR transcriptional target that compromised rapamycin responsiveness when either the C or G was mutated (115) Thus, it is tempting to speculate that CREBRF and REPTOR may bind to a methylated CpG-containing site in a ZEBRA-like manner.

SUPPLEMENTARY REFERENCES

118. Reinke, A.W., Grant, R.A. and Keating, A.E. (2010) A synthetic coiled-coil interactome provides heterospecific modules for molecular engineering. *J Am Chem Soc*, **132**, 6025-6031.

119. Young, L.S., Yap, L.F. and Murray, P.G. (2016) Epstein-Barr virus: more than 50 years old and still providing surprises. *Nat Rev Cancer*, **16**, 789-802.

120. Audas, T.E., Li, Y., Liang, G. and Lu, R. (2008) A novel protein, Luman/CREB3 recruitment factor, inhibits Luman activation of the unfolded protein response. *Mol Cell Biol*, **28**, 3952-3966.

121. Martyn, A.C., Choleris, E., Gillis, D.J., Armstrong, J.N., Amor, T.R., McCluggage, A.R., Turner, P.V., Liang, G., Cai, K. and Lu, R. (2012) Luman/CREB3 recruitment factor regulates glucocorticoid receptor activity and is essential for prolactin-mediated maternal instinct. *Mol Cell Biol*, **32**, 5140-5150.

122. Audas, T.E., Hardy-Smith, P.W., Penney, J., Taylor, T. and Lu, R. (2016) Characterization of nuclear foci-targeting of Luman/CREB3 recruitment factor (LRF/CREBRF) and its potential role in inhibition of herpes simplex virus-1 replication. *Eur J Cell Biol*, **95**, 611-622.

123. Xue, H., Zhang, J., Guo, X., Wang, J., Li, J., Gao, X., Guo, X., Li, T., Xu, S., Zhang, P. *et al.* (2016) CREBRF is a potent tumor suppressor of glioblastoma by blocking hypoxia-induced autophagy via the CREB3/ATG5 pathway. *Int J Oncol*, **49**, 519-528.

124. Minster, R.L., Hawley, N.L., Su, C.T., Sun, G., Kershaw, E.E., Cheng, H., Buhule, O.D., Lin, J., Reupena, M.S., Viali, S. *et al.* (2016) A thrifty variant in CREBRF strongly influences body mass index in Samoans. *Nat Genet*, **48**, 1049-1054.

125. Han, J., Zhang, L., Zhang, J., Jiang, Q., Tong, D., Wang, X., Gao, X., Zhao, L. and Huang, C. (2018) CREBRF promotes the proliferation of human gastric cancer cells via the AKT signaling pathway. *Cell Mol Biol*, **64**, 40-45.
